# Entropy optimization of lid-driven micropolar hybrid nanofluid flow in a partially porous hexagonal-shaped cavity with relevance to energy efficient storage processes

**DOI:** 10.1038/s41598-024-60483-y

**Published:** 2024-04-27

**Authors:** Anil Ahlawat, Shilpa Chaudhary, Mukesh Kumar Sharma, K. Loganathan, Balachandra Pattanaik, Allam Balaram

**Affiliations:** 1 Department of Mathematics & Statistics, Manipal University Jaipur, Jaipur, 303007 Rajasthan India; 2 Department of Mathematics, Guru Jambheshwar University of Science and Technology Hisar, Hisar, 125001 Haryana India; 3 School of Electrical and Computer Engineering, Wollega University, P.O.BOX:395, Nekemte, Ethiopia; 4 Department of Computer Science and Engineering, MLR Institute of Technology, Hyderabad, Telangana India

**Keywords:** Porous medium, Hexagonal enclosure, Hybrid nanofluid, Lid-driven flow, Mathematics and computing, Applied mathematics

## Abstract

Hydromagnetically associated heat convection can greatly enhance the performance of high-efficiency thermal appliances and renewable energy sources through an optimized design. This investigation examines the production of thermodynamic irreversibility and heat convection for a double lid-driven flow within a partially porous stratified hexagonal enclosure. The top and bottom-wall are moving in the opposite direction with an equal velocity U_0_. The top-wall and the bottom-wall are kept at temperature T_c_ and T_h_ (T_h_ $>$ T_c_) while the slanted walls are assumed to be thermally insulated. A constant magnetic field is employed in the horizontal x-direction. The hexagonal cavity was filled with a micropolar hybrid nanofluid Ag-MgO/water. The system of dimensionless equations was solved by the finite difference method (FDM) associated with successive over-relaxation (SOR), successive under-relaxation (SUR), and Gauss–Seidel iteration tactics and required results are computed with problem specific program in MATLAB code. The results indicate that the Ra and the thickness of the porous layer (X_p_) significantly influences heat convection and thermal irreversibility processes. The Nu_avg_ and S_Total_ rises 6.299% and 3.373% as ‘${\upphi }_{{\text{hnf}}}$’ enhances from 0 to 4%, respectively. Furthermore, as the values of Ra, Ha, K_0_, and ${\upphi }_{{\text{hnf}}}$ increase, Be_avg_ experiences a decline of 53.73%, 11.04%, 38.36%, and 0.09% respectively. Also, movement of wall has a significant impact on heat transfer rates and entropy production. The present study may be extended in numerous areas to mimic the problems like—(1) onset of thermo-mechanical process for solid–fluid interaction in a conduit. (2) Thermos-chemical process with extraction of ions in two-phase fluid for double layer plating on a continuously moving sheet, as region of porous stratum saturated with a class of fluid and region without porous medium occupied with other fluid.

## Introduction

The worldwide growth in renewable energy demands depends on improved efficiency of heat transfer as well as a diminution of energy waste. Advancing the engineering of innovative power devices and upgrading sophisticated scientific processes can significantly boost energy production to meet growing demand. It is worth mentioning that there has been a significant surge in research efforts focused on advancing heat transfer methods. The nanoscale technology has garnered the interest of numerous investigators due to its potential to improve heat transmission in industrial processes and minimize the production of excessive heat. The introduction of nanofluids (NFs) manifests a promising approach for augmenting heat conveyance in addition to designing heat exchanger geometries. According to Choi^1^, “NFs is a sophisticated mixture that combines a conventional fluid having poor thermal conductivity together with nano-sized particles of metal or metal oxide.” Recently, NFs have become more and more vital for the development of high-efficiency thermal appliances and have been significantly documented in studies by Das et al.^2^, Murshed et al.^3^, Sarkar^4^, and Yu and Xie^5^. NFs have been employed in manufacturing-related sectors, as outlined in the work of Wong and Leon^6^. An entirely novel category of NFs called hybrid nanofluids (HNFs) emerged as a result of the synthesis of NFs; these HNFs incorporate two different kinds of nanoparticles. Initially, Jana et al.^7^ performed qualitative research concerning this specific category of NFs. Novel HNFs has the potential to achieve enhanced thermal conductivity, outperforming single nanoparticle-based nanofluids. The works published by Esfe et al.^8^, Jamei and Ahmadianfar^9^, Rashidi et al.^10^, and Madhesh and Kalaiselvam^11^ contribute to a comprehensive collection of research on the evaluation of HNFs in terms of their viscosity and thermal efficiency, in addition to other physical characteristics.

The study of thermal mechanisms in a space with moving walls has garnered significant attention. This approach is highly adaptable and extensively utilized in electronics and related areas. This type of structure is known as a lid-driven cavity in terms of engineering. Few studies have focused on this specific concern; most have only examined situations with one or two moving walls. In the past decade, investigators, especially Talebi et al.^12^, Aminossadati et al.^13^, Fattahi et al.^14^, Salari et al.^15^, Cho and Chen^16^, Muthtamilselvan and Doh^17^, Abu-Nada and Chamkha^18^, Kefayati^19^, and Oztop et al.^20^, have conducted numerical investigations on the phenomenon of lid-driven mixed convection in enclosures utilizing various structures and boundary constraints. Al Kalbani et al. ^21^ examined the impact of magnetic fields on the occurrence of free convective flow in a cavity comprising six different types of nanofluids. Manna et al.^22^ examined the impact of a multibanded magnetic field on convective heat transmission in a linearly heated porous system utilizing hybrid nanofluid and discovered that the multibanded magnetic field is a promising approach for controlling convective transport phenomena in complex applications that involve coupled multiphysics. Biswas et al.^23^ examined the impact of surface waviness on MHD thermo-gravitational convection of a Cu–Al_2_O_3_–water hybrid nanofluid in a porous oblique enclosure and found that, even when the effective heating surface area increases, a wavy curved wall does not necessarily ensure an improvement in heat transfer. Biswas et al.^24^ highlighted the utilization of a partial magnetic field in a conventional thermal system. Biswas et al.^25^ shows that partially active magnetic fields have a positive effect on the thermal performance of hybrid nanofluid (Cu–Al_2_O_3_–H_2_O) flow in an oblique wavy porous enclosure. They found that a partially active magnetic field can effectively control field variables with less reduction in the transfer of heat compared to using a magnetic field throughout the entire domain. Mondal et al.^26^ conducted a study on the thermal stability of hybrid nanofluid flow in a slanted porous enclosure by applying partial magnetic fields. The analysis reveals that the presence of partial magnetic fields with cavity orientation significantly impacts the heat transmission process. Hamzah et al.^27^ simulated a wavy lid on the conduction of heat and entropy production in a porous enclosure saturated with CNT-water nanofluid under exposure to a magnetic field. Digital models of lid-driven cavities with staircases trapped in the center were created by Huang and Lim^28^. The findings obtained demonstrate the correlation between the motion of the wall and the deformation of the fluid constituents. In addition, the study focuses on investigating the impact of obstacle size on the resulting flow. A porous substrate is characterized by its intricate network of microscopic paths, commonly referred to as pores, through which liquids can pass. The academic and industrial communities have long been concerned with finding ways to maximize the effectiveness and efficiency of energy consumption. Materials with pores frequently enhance heat transmission. In their studies, Poulikakos et al.^29^ and Beckermann et al.^30^ examined heat transfer in a liquid and an overlying porous layer that is either horizontally or vertically positioned and has an interface that is either permeable or impermeable. Vafai^31^ examined diverse applications of porous materials, including heat exchangers for transmitting heat from gadgets to biological structures, geothermal engineering, oil well engineering, thermal blankets, and other fields. According to the work of Mohamad^32^ and Maerefat et al.^33^, heat convection in systems that are partially filled with a porous medium has the benefit of minimizing pressure drop in contrast to systems that are entirely filled with the porous material. Rong et al.^34^ investigated the influence of porous material on an upsurge in heat transmission in a pipe flow and found that the addition of a porous layer significantly enhances heat transfer, and this enhancement is directly proportional to the thickness of the porous layer. Researchers Sun and Pop^35^, Chamkha and Ismael^36^, and Sheremet et al.^37^ have studied the flow of nanofluids in chambers partially or entirely filled with porous media. In a recent study, Ahlawat and Sharma^38^ investigated how the thickness of the porous layer influenced the generation of entropy and the transmission of heat in a closed system with a heated block at its center. Effective utilization of accessible energy necessitates the minimization of entropy generation. The “second law of thermodynamics” suggests that the failure of an electrical device due to high temperatures can be prevented by improving the process of heat transfer within the device. Bejan^39,40^ concentrated on the evaluation of generated entropy as an attempt to determine the correlation between “thermodynamics, heat transfer, and fluid mechanics.” Baytas^41^ conducted a numerical study on the production of entropy in a two-dimensional smooth flow inside a slanted permeable enclosure, utilizing Darcy’s law. Rashad et al.^42^, Mourad et al.^43^, Abdel-Nour et al.^44^, Armaghani et al.^45^, and Ahlawat and Sharma^46^ conducted quantitative investigations to analyze the production of entropy and the conveyance of heat under varying conditions in various chambers. A diversity of scientific fields has discovered novel and intriguing applications for “non-Newtonian fluids” due to their unique rheological properties, including in technology, industry, biological processes, etc.

“Classical Theory of Continuum Mechanics,” introduced by Navier’s Stokes model, fails to provide an explanation for fluids having microstructures. “Microcontinuum theories” emerged as a result of the limitations of “classical continuum theory,” which assumes that continuous media possess mass, velocity, and microstructure. The examination of the distortion, shape, and inherent movement of particular suspended constituents has paved the way for the advancement of various novel theories, such as the “theory of simple microfluids” as outlined by Eringen^47^. Micropolar fluids (MFs) are “a particular class of non-Newtonian fluids” described as mixtures of randomly dispersed solid constituents in a fluid where the deformation of the constituents is disregarded. In their study, Papautsky et al.^48^ initially examined the relationship between computational and experimental findings for laminar flow implementing the micropolar fluid theory and found that the Darcy friction factor boosts when working with MFs, which is contrary to the findings derived from the Navier’s-Stokes theory. As reported by Tayebi et al. ^49^, the rate of heat transfer in a system with two heated cylinders comprising a micropolar Al_2_O_3_-water nanofluid increases with a higher Rayleigh number and decreases with a larger vortex viscosity (K). Ahlawat and Sharma^50^ performed recent research on a convective heat transfer mechanism that makes use of discrete heaters within an annulus.

The hexagonal enclosure’s strength as a heat sink and its application in the chemical industry as micromixers have brought it global recognition. The idea of enclosing a partially porous structure and a partially liquid-filled chamber with movable upper and lower walls has many potential practical uses in many fields. For example, water filtration systems; biomedical devices; chemical reactors; fuel cells; heat exchangers; microfluidic devices; environmental remediation; and the oil and gas industry. The previous research suggests that the current study aims to address a knowledge gap by examining the impact of double lid-driven flow on entropy production and heat transfer within a partially porous hexagonal cavity, saturated with micropolar hybrid nanofluid and exposed to a transverse magnetic field. The key goal of this study is to optimize heat transfer efficiency and maximize energy utilization in thermal gadgets by modifying nanofluid technology. The outcomes of the study provide a significant contribution to the discipline of thermal science and engineering, aiding in the advancement of efficient heat-transfer devices that utilize HNFs.

## Problem formulation

**Figure 1:**
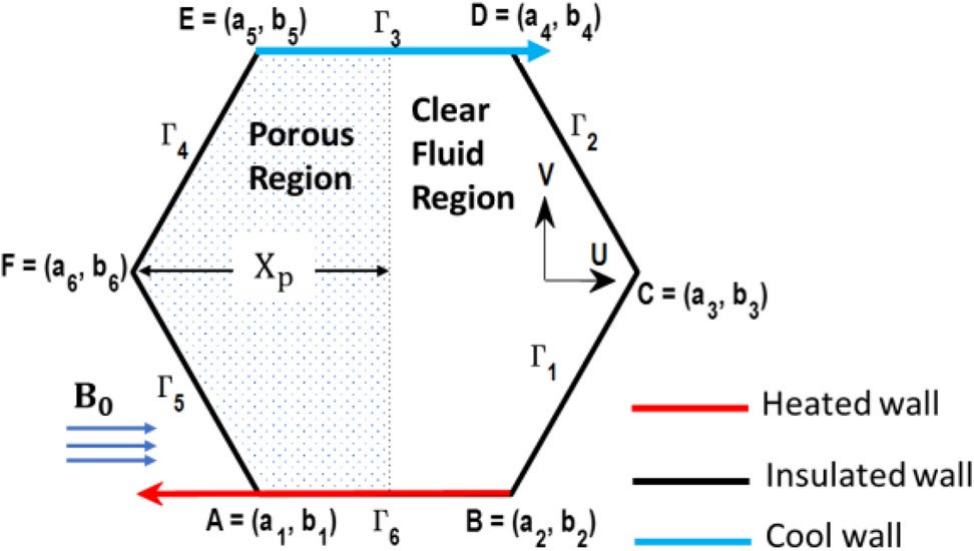
Schematic representation of the problem being analyzed.

Figure 1 depicts the assessed hexagonal enclosure. The upper wall is moving horizontally in the positive x direction at a uniform velocity of U_0_, while the lower wall is moving in the opposite direction with an equal velocity. Table 1 provides a detailed listing of the hexagonal vertices, which are located at A, B, C, D, E, and F. The characters ${\Gamma }_{1}$, ${\Gamma }_{2}$, ${\Gamma }_{3}$, ${\Gamma }_{4}$, ${\Gamma }_{5}$, and ${\Gamma }_{6}$ are used to indicate the boundaries of the hexagonal enclosure. The top wall is kept at a lower temperature T_c_ and the bottom wall are at high temperature T_h_ i.e., T_h_ $>$ T_c_ while the slanted walls are assumed to be thermally insulated. A static magnetic field of intensity ‘B_0_’ is employed in the x-direction. The hexagonal enclosure is partitioned into two layers. The right layer contains a clear hybrid nanofluid (Ag-MgO/H_2_O), while the left layer is stuffed with a porous medium having a thickness ‘X_p_’ that is entirely occupied utilizing the same hybrid nanofluid. Darcy’s law to describe the porous medium’s characteristics and the Boussinesq approximation for density variation were employed. “The solid matrix in the porous layer and nanoparticles,” are assumed to be in a state of local thermal equilibrium. Table 2 displays the thermophysical characteristics of the constituents of hybrid nanofluid.

**Table 1:** The specified configuration of edges for the hexagonal cavity.

A	(a_1_, b_1_) = (0.25H, 0)
B	(a_2_, b_2_) = (0.75H, 0)
C	(a_3_, b_3_) = (H, 0.50H)
D	(a_4_, b_4_) = (0.75H, H)
E	(a_5_, b_5_) = (0.25H, H)
F	(a_6_, b_6_) = (0, 0.50H)

**Table 2:** Thermophysical characteristics of ${{\text{H}}}_{2}{\text{O}}$, MgO and Ag as given in Ghalambaz et al.^53^.

Physical parameter	C_p_ (J kg^–1^ K^–1^)	ρ (Kg m^–3^)	k (W m^–1^ K^–1^)	β (K^–1^)	σ (Ω^–1^ m^–1^)
${{\text{H}}}_{2}{\text{O}}$	4179	997.1	0.613	21 × 10^–5^	5.5 × 10^–6^
MgO	879	3580	30	3.36 × 10^–5^	8 × 10^–4^
Ag	235	10,500	429	5.4 × 10^–5^	8.1 × 10^–4^

In light of the work of “Eringen^47,51^ and Mansour et al.^52^”, “continuity, linear momentum, angular momentum, and energy equations” are formulated to represent laminar, steady-state micropolar fluid flow, taking advantage of the premises that were mentioned above:

**For Porous layer:**

1 $$\frac{{\partial {\text{u}}_{{\text{p}}} }}{{\partial {\text{x}}}} + \frac{{\partial {\text{v}}_{{\text{p}}} }}{{\partial {\text{y}}}} = 0$$

2 $${\uprho }_{{{\text{hnf}}}} \left( {{\text{u}}_{{\text{p}}} \frac{{\partial {\text{u}}_{{\text{p}}} }}{{\partial {\text{x}}}} + {\text{v}}_{{\text{p}}} \frac{{\partial {\text{u}}_{{\text{p}}} }}{{\partial {\text{y}}}}} \right) = - \in^{2} \frac{{\partial {\text{p}}}}{{\partial {\text{x}}}} + \in \left( {{\upmu }_{{{\text{hnf}}}} + {\upkappa }} \right)\left( {\frac{{\partial^{2} {\text{u}}_{{\text{p}}} }}{{\partial {\text{x}}^{2} }} + \frac{{\partial^{2} {\text{u}}_{{\text{p}}} }}{{\partial {\text{y}}^{2} }}} \right) + \in^{2} {\upkappa }\frac{{\partial {\text{m}}_{{\text{p}}} }}{{\partial {\text{y}}}} - \in^{2} \frac{{{\upmu }_{{{\text{hnf}}}} }}{{\text{K}}}{\text{u}}_{{\text{p}}}$$

3 $$\begin{aligned} {{\rho }}_{{{\text{hnf}}}} \left( {{\text{u}}_{{\text{p}}} \frac{{\partial {\text{v}}_{{\text{p}}} }}{{\partial {\text{x}}}} + {\text{v}}_{{\text{p}}} \frac{{\partial {\text{v}}_{{\text{p}}} }}{{\partial {\text{y}}}}} \right) & = - \in ^{2} \frac{{\partial {\text{p}}}}{{\partial {\text{y}}}} + \in \left( {{{\mu }}_{{{\text{hnf}}}} + {{\kappa }}} \right)\left( {\frac{{\partial ^{2} {\text{v}}_{{\text{p}}} }}{{\partial {\text{x}}^{2} }} + \frac{{\partial ^{2} {\text{v}}_{{\text{p}}} }}{{\partial {\text{y}}^{2} }}} \right) - \in ^{2} {{\kappa }}\frac{{\partial {\text{m}}_{{\text{p}}} }}{{\partial {\text{x}}}} \\ & \;\;\; - \in ^{2} \frac{{{{\mu }}_{{{\text{hnf}}}} }}{{\text{K}}}{\text{v}}_{{\text{p}}} + \in ^{2} \left( {{{\rho \beta }}} \right)_{{{\text{hnf}}}} ~{{\vec{\rm g}}}\left( {{\text{T}}_{{\text{p}}} - {\text{T}}_{{\text{c}}} } \right) + \in ^{2} {{\sigma }}_{{{\text{hnf}}}} {\text{~B}}_{0}^{2} {\text{v}}_{{\text{p}}} \\ \end{aligned}$$

4 $$\left( {{\text{u}}_{{\text{p}}} \frac{{\partial {\text{T}}_{{\text{p}}} }}{{\partial {\text{x}}}} + {\text{v}}_{{\text{p}}} \frac{{\partial {\text{T}}_{{\text{p}}} }}{{\partial {\text{y}}}}} \right) = {\upalpha }_{{{\text{eff}}}} \left( {\frac{{\partial^{2} {\text{T}}_{{\text{p}}} }}{{\partial {\text{x}}^{2} }} + \frac{{\partial^{2} {\text{T}}_{{\text{p}}} }}{{\partial {\text{y}}^{2} }}} \right)$$

5 $${\uprho }_{{{\text{hnf}}}} \left( {{\text{u}}_{{\text{p}}} \frac{{\partial {\text{m}}_{{\text{p}}} }}{{\partial {\text{x}}}} + {\text{v}}_{{\text{p}}} \frac{{\partial {\text{m}}}}{{\partial {\text{y}}}}} \right) = \in \frac{{{\upgamma }_{{{\text{hnf}}}} }}{{\text{j}}}\left( {\frac{{\partial^{2} {\text{m}}_{{\text{p}}} }}{{\partial {\text{x}}^{2} }} + \frac{{\partial^{2} {\text{m}}_{{\text{p}}} }}{{\partial {\text{y}}^{2} }}} \right) - \in \frac{{2{\upkappa }}}{{\text{j}}}{\text{m}}_{{\text{p}}} + \frac{{\upkappa }}{{\text{j}}}\left( {\frac{{\partial {\text{v}}_{{\text{p}}} }}{{\partial {\text{x}}}} - \frac{{\partial {\text{u}}_{{\text{p}}} }}{{\partial {\text{y}}}}} \right)$$

**For hybrid nanofluid layer:**

6 $$\frac{{\partial {\text{u}}_{{{\text{hnf}}}} }}{{\partial {\text{x}}}} + \frac{{\partial {\text{v}}_{{{\text{hnf}}}} }}{{\partial {\text{y}}}} = 0$$

7 $${\uprho }_{{{\text{hnf}}}} \left( {{\text{u}}_{{{\text{hnf}}}} \frac{{\partial {\text{u}}_{{{\text{hnf}}}} }}{{\partial {\text{x}}}} + {\text{v}}_{{{\text{hnf}}}} \frac{{\partial {\text{u}}_{{{\text{hnf}}}} }}{{\partial {\text{y}}}}} \right) = - \frac{{\partial {\text{p}}}}{{\partial {\text{x}}}} + \left( {{\upmu }_{{{\text{hnf}}}} + {\upkappa }} \right)\left( {\frac{{\partial^{2} {\text{u}}_{{{\text{hnf}}}} }}{{\partial {\text{x}}^{2} }} + \frac{{\partial^{2} {\text{u}}_{{{\text{hnf}}}} }}{{\partial {\text{y}}^{2} }}} \right) + {\upkappa }\frac{{\partial {\text{m}}_{{{\text{hnf}}}} }}{{\partial {\text{y}}}}$$

8 $$\begin{aligned} {\uprho }_{{{\text{hnf}}}} \left( {{\text{u}}_{{{\text{hnf}}}} \frac{{\partial {\text{v}}_{{{\text{hnf}}}} }}{{\partial {\text{x}}}} + {\text{v}}_{{{\text{hnf}}}} \frac{{\partial {\text{v}}_{{{\text{hnf}}}} }}{{\partial {\text{y}}}}} \right) & = - \frac{{\partial {\text{p}}}}{{\partial {\text{y}}}} + \left( {{\upmu }_{{{\text{hnf}}}} + {\upkappa }} \right)\left( {\frac{{\partial^{2} {\text{v}}_{{{\text{hnf}}}} }}{{\partial {\text{x}}^{2} }} + \frac{{\partial^{2} {\text{v}}_{{{\text{hnf}}}} }}{{\partial {\text{y}}^{2} }}} \right) - {\upkappa }\frac{{\partial {\text{m}}_{{{\text{hnf}}}} }}{{\partial {\text{x}}}} \\ & \;\;\; + {\upsigma }_{{{\text{hnf}}}} {\text{ B}}_{0}^{2} {\text{v}}_{{{\text{hnf}}}} + \left( {{{\rho \beta }}} \right)_{{{\text{hnf}}}} {\vec{\text{g}}}\left( {{\text{T}}_{{{\text{hnf}}}} - {\text{T}}_{{\text{c}}} } \right) \\ \end{aligned}$$

9 $$\left( {{\text{u}}_{{{\text{hnf}}}} \frac{{\partial {\text{T}}_{{{\text{hnf}}}} }}{{\partial {\text{x}}}} + {\text{v}}_{{{\text{hnf}}}} \frac{{\partial {\text{T}}_{{{\text{hnf}}}} }}{{\partial {\text{y}}}}} \right) = {\upalpha }_{{{\text{hnf}}}} \left( {\frac{{\partial^{2} {\text{T}}_{{{\text{hnf}}}} }}{{\partial {\text{x}}^{2} }} + \frac{{\partial^{2} {\text{T}}_{{{\text{hnf}}}} }}{{\partial {\text{y}}^{2} }}} \right)$$

10 $${\uprho }_{{{\text{hnf}}}} \left( {{\text{u}}_{{{\text{hnf}}}} \frac{{\partial {\text{m}}_{{{\text{hnf}}}} }}{{\partial {\text{x}}}} + {\text{v}}_{{{\text{hnf}}}} \frac{{\partial {\text{m}}_{{{\text{hnf}}}} }}{{\partial {\text{y}}}}} \right) = \frac{{{\upgamma }_{{{\text{hnf}}}} }}{{\text{j}}}\left( {\frac{{\partial^{2} {\text{m}}_{{{\text{hnf}}}} }}{{\partial {\text{x}}^{2} }} + \frac{{\partial^{2} {\text{m}}_{{{\text{hnf}}}} }}{{\partial {\text{y}}^{2} }}} \right) - \frac{{2{\upkappa }}}{{\text{j}}}{\text{m}}_{{{\text{hnf}}}} + \frac{{\upkappa }}{{\text{j}}}\left( {\frac{{\partial {\text{v}}_{{{\text{hnf}}}} }}{{\partial {\text{x}}}} - \frac{{\partial {\text{u}}_{{{\text{hnf}}}} }}{{\partial {\text{y}}}}} \right)$$

Hybrid nanofluid’s thermophysical properties in relation to volume concentration of nanoparticles ($\phi_{{{\text{Ag}}}} \;\;{\text{and}}\;\;{ }\phi_{{{\text{MgO}}}}$) are used as described in Eqs. (11–13) as follows, Ghalambaz et al.^53^:

11 $$\left.\begin{array}{c}{\upphi }_{{\text{hnf}}}={\upphi }_{{\text{Ag}}}+{\upphi }_{\mathrm{MgO }} \\ {\uprho }_{{\text{hnf}}}=\left(1-{\upphi }_{{\text{hnf}}}\right){\uprho }_{{\text{f}}}+{\upphi }_{{\text{Ag}}}{\uprho }_{{\text{Ag}}}+{\upphi }_{{\text{MgO}}}{\uprho }_{{\text{MgO}}} \\ {\left(\mathrm{\rho \beta }\right)}_{{\text{hnf}}}=\left(1-{\upphi }_{{\text{hnf}}}\right){\uprho }_{{\text{f}}}+{\upphi }_{{\text{Ag}}}{\left(\mathrm{\rho \beta }\right)}_{{\text{Ag}}}+{\upphi }_{{\text{MgO}}}{\left(\mathrm{\rho \beta }\right)}_{{\text{MgO}}} \\ {\left(\uprho {{\text{C}}}_{{\text{p}}}\right)}_{{\text{hnf}}}=\left(1-{\upphi }_{{\text{hnf}}}\right){\uprho }_{{\text{f}}}+{\upphi }_{{\text{Ag}}}{\left(\uprho {{\text{C}}}_{{\text{p}}}\right)}_{{\text{Ag}}}+{\upphi }_{{\text{MgO}}}{\left(\uprho {{\text{C}}}_{{\text{p}}}\right)}_{{\text{MgO}}} \\ {\mathrm{\alpha }}_{{\text{hnf}}}=\frac{{{\text{k}}}_{{\text{hnf}}}}{{\left(\uprho {{\text{C}}}_{{\text{p}}}\right)}_{{\text{hnf}}}} ; {\mathrm{\alpha }}_{{\text{eff}}}=\frac{{{\text{k}}}_{{\text{eff}}}}{{\left(\uprho {{\text{C}}}_{{\text{p}}}\right)}_{{\text{hnf}}}} where {{\text{k}}}_{{\text{eff}}}=\left(1-\upvarepsilon \right){{\text{k}}}_{{\text{s}}}+\varepsilon {{\text{k}}}_{{\text{hnf}}}\end{array}\right\}$$

where “$\in$—the porosity of the porous stratum, ${\beta }$—the thermal expansion coefficient, ${\uprho }_{{{\text{hnf}}}}$—the density of hybrid nanofluid, ${\text{K }}$—the permeability of the porous stratum, ${\upmu }_{{{\text{hnf}}}}$—the dynamic viscosity of hybrid nanofluid and ${\upalpha }_{{{\text{hnf}}}}$—the thermal diffusivity. The ‘${\text{p}}$’ and ‘${\text{hnf}}$’ are subscripts that stand for porous medium and hybrid nanofluid. $\left( {{\rho C}_{{\text{p}}} } \right)_{{{\text{hnf}}}}$—the heat capacity of hybrid nanofluid. ${\text{k}}_{{{\text{hnf}}}}$—thermal conductivity of hybrid nanofluids”. The current investigation employs the “Maxwell model” ^54^ to describe the ‘${\text{k}}_{{{\text{hnf}}}}$’ and the corresponding expression is as follows:

12 $${\text{k}}_{{{\text{hnf}}}} = \left[ {\frac{{\left( {\frac{{\phi_{{{\text{Ag}}}} {\text{k}}_{{{\text{Ag}}}} + \phi_{{{\text{MgO}}}} {\text{k}}_{{{\text{MgO}}}} }}{{\phi_{{{\text{hnf}}}} }} + 2{\text{k}}_{{\text{f}}} } \right) + 2\left( {\phi_{{{\text{Ag}}}} {\text{k}}_{{{\text{Ag}}}} + \phi_{{{\text{MgO}}}} {\text{k}}_{{{\text{MgO}}}} } \right) - 2\phi_{{{\text{hnf}}}} {\text{k}}_{{\text{f}}} }}{{\left( {\frac{{\phi_{{{\text{Ag}}}} {\text{k}}_{{{\text{Ag}}}} + \phi_{{{\text{MgO}}}} {\text{k}}_{{{\text{MgO}}}} }}{{\phi_{{{\text{hnf}}}} }} + 2{\text{k}}_{{\text{f}}} } \right) - \left( {\phi_{{{\text{Ag}}}} {\text{k}}_{{{\text{Ag}}}} + \phi_{{{\text{MgO}}}} {\text{k}}_{{{\text{MgO}}}} } \right) + \phi_{{{\text{hnf}}}} {\text{k}}_{{\text{f}}} }}} \right]{\text{k}}_{{\text{f}}}$$

The viscosity of hybrid nanofluids (${\upmu }_{{{\text{hnf}}}}$) is determined using the following Eq. (13), which is based on the Brinkman model^55^.

13 $${\upmu }_{{{\text{hnf}}}} = \frac{{{\upmu }_{{\text{f}}} }}{{\left( {1 - \phi_{{{\text{hnf}}}} } \right)^{2.5} }}$$

**Dimensional boundary conditions are determined and classified as:**

The upper (${\Gamma }_{3}$) and lower (${\Gamma }_{6}$) walls have the condition, u = U_0_; v = m = 0; T = T_c_ and u = -U_0_; v = m = 0; T = T_h_, respectively. Here

14 $$\left. {\begin{array}{*{20}c} {{\Gamma }_{3} = \left\{ {\left( {{\text{x}},{\text{y}}} \right) \in {\text{R}}^{2} :{\text{y}} = {\text{H}};\;\;{\text{where}}\;{ }0.25{\text{H}} \le {\text{x}} \le 0.75{\text{H}}} \right.} \\ {{\Gamma }_{6} = \left\{ {\left( {{\text{x}},{\text{y}}} \right) \in {\text{R}}^{2} :{\text{y}} = 0;\;\;{\text{where }}\;0.25{\text{H}} \le {\text{x}} \le 0.75{\text{H}}} \right.} \\ \end{array} } \right\}$$

The conditions associated with slanted walls ${\Gamma }_{1}$, ${\Gamma }_{2}$, ${\Gamma }_{4}$, ${\Gamma }_{5}$ are specified as follows:

u = v = m = 0; $\frac{\partial T}{{\partial n}} = 0$. Where ‘*n*’ is the unit vector.

15 $$\left.\begin{array}{c}{\Gamma }_{1}=\left\{\left({\text{x}},{\text{y}}\right)\in {{\text{R}}}^{2}:{\text{y}}=2\left({\text{x}}-\frac{3}{4}{\text{H}}\right);\mathrm{where }~0.75{\text{H}}\le {\text{x}}\le {\text{H}}\right. \\ {\Gamma }_{2}=\left\{\left({\text{x}},{\text{y}}\right)\in {{\text{R}}}^{2}:{\text{y}}-\frac{H}{2}=-2({\text{x}}-{\text{H}});\mathrm{where }~0.75{\text{H}}\le {\text{x}}\le {\text{H}}\right.\\ {\Gamma }_{4}=\left\{\left({\text{x}},{\text{y}}\right)\in {{\text{R}}}^{2}:{\text{y}}-{\text{H}}=2\left({\text{x}}-\frac{H}{4}\right);\mathrm{where }~0\le {\text{x}}\le 0.25\mathrm{H }\right.\\ {\Gamma }_{5}=\left\{\left({\text{x}},{\text{y}}\right)\in {{\text{R}}}^{2}:{\text{y}}-\frac{H}{2}=-2{\text{x}};\mathrm{where } ~0\le {\text{x}}\le 0.25{\text{H}}\right.\end{array}\right\}$$

The following non-dimensional parameters described in Eq. (16) are used in the Eqs. (1–10) to obtain the dimensionless governing Eqs. (15)–(18):

16 $$\begin{aligned} {\text{X}} & = \frac{{\text{x}}}{{\text{H}}};{\text{Y}} = \frac{{\text{y}}}{{\text{H}}};{{ \uptheta }}_{{{\text{hnf}}}} = \frac{{{\text{T}}_{{{\text{hnf}}}} - {\text{T}}_{{\text{c}}} }}{{{\Delta T}}};{{ \uptheta }}_{{\text{p}}} = \frac{{{\text{T}}_{{\text{p}}} - {\text{T}}_{{\text{c}}} }}{{{\Delta T}}};{\text{Ra}} = \frac{{{{{\rm g}\beta }}_{{\text{f}}} {{\Delta {\rm TH}}}^{3} }}{{{\upnu }_{{\text{f}}} {\upalpha }_{{\text{f}}} }};{\text{Re}} = \frac{{{\text{U}}_{0} {\text{H}}}}{{{\upnu }_{{\text{f}}} }}; \\ {\text{Da}} & = \frac{{\text{K}}}{{{\text{H}}^{2} }};{{ \chi }} = \frac{{{\text{H}}^{2} }}{{\text{j}}};{\text{ K}}_{0} = \frac{{\text{k}}}{{{\upmu }_{{\text{f}}} }};{\text{M}}_{{\text{p}}} = \frac{{{\text{m}}_{{\text{p}}} {\text{H}}^{2} }}{{{\upalpha }_{{\text{f}}} }};{\text{ M}}_{{{\text{hnf}}}} = \frac{{{\text{m}}_{{{\text{hnf}}}} {\text{H}}^{2} }}{{{\upalpha }_{{\text{f}}} }}{ };{\text{ U}}_{{\text{p}}} = \frac{{{\text{u}}_{{\text{p}}} }}{{{\text{U}}_{0} }};{\text{V}}_{{\text{p}}} = \frac{{{\text{v}}_{{\text{p}}} }}{{{\text{U}}_{0} }}; \\ {\text{U}}_{{{\text{hnf}}}} & = \frac{{{\text{u}}_{{{\text{hnf}}}} }}{{{\text{U}}_{0} }};{\text{V}}_{{{\text{hnf}}}} \frac{{{\text{v}}_{{{\text{hnf}}}} }}{{{\text{U}}_{0} }}{ };{\text{P}} = \frac{{\text{p}}}{{{\uprho }_{{\text{f}}} {\text{U}}_{0}^{2} }};{\upchi } = \frac{{{\text{H}}^{2} }}{{\text{j}}} \\ \end{aligned}$$

$\Delta {\text{T}}$  =  ${\text{T}}_{{\text{h}}} { }{-}{\text{T}}_{{\text{c}}}$. Where, “Pr, Da, ${\upkappa }$, K_0_,${{ \chi }}$, ${\text{J}}$, Re and Ra are Prandtl number, Darcy number, vortex viscosity parameter, dimensionless vortex viscosity parameter, material parameter, micro-inertial density, Reynolds number and Rayleigh number”, respectively. Taking the spin-gradient viscosity, ${\upgamma }_{{{\text{hnf}}}} = \left( {{\upmu }_{{{\text{hnf}}}} + \frac{{\upkappa }}{2}} \right){\text{j}}$ as defined in Ahmadi^56^; Rees and Pop^57^ and implementing the “stream function approach” with ${\text{U}} = \frac{{\partial {\uppsi }}}{{\partial {\text{Y}}}}$, ${\text{V}} = - \frac{{\partial {\uppsi }}}{{\partial {\text{X}}}}$, $= \frac{{\partial {\text{V}}}}{{\partial {\text{X}}}} - \frac{{\partial {\text{U}}}}{{\partial {\text{Y}}}}$ the dimensionless equations, jointly for the porous and fluid layers, are derived through Eqs. (17–20).

17 $$\left( {\frac{{\partial^{2} }}{{\partial {\text{X}}^{2} }} + \frac{{\partial^{2} }}{{\partial {\text{Y}}^{2} }}} \right){\uppsi }_{{{\text{p}},{\text{ hnf}}}} = - {\upomega }_{{{\text{p}},{\text{ hnf}}}}$$

**Vorticity equation:**

18 $$\begin{aligned} \left( {{\text{U}}_{{{\text{p}},{\text{hnf}}}} \frac{\partial }{{\partial {\text{X}}}} + {\text{V}}_{{{\text{p}},{\text{hnf}}}} \frac{\partial }{{\partial {\text{Y}}}}} \right){{\omega }}_{{{\text{p}},{\text{hnf}}}} & = {{\varepsilon ~}}\left( {\frac{1}{{{\text{Re}}}}} \right)\left( {\frac{{{{\rho }}_{{\text{f}}} }}{{{{\rho }}_{{{\text{hnf}}}} }}} \right)\left( {\frac{1}{{\left( {1 - \phi _{{{\text{hnf}}}} } \right)^{{2.5}} }} + {\text{K}}_{0} } \right)\left( {\frac{{\partial ^{2} }}{{\partial {\text{X}}^{2} }} + \frac{{\partial ^{2} }}{{\partial {\text{Y}}^{2} }}} \right){{\omega }}_{{{\text{p}},{\text{hnf}}}} \\ & \;\;\; - {{\varepsilon }}^{2} {\text{K}}_{0} {\text{~}}\left( {\frac{{{{\rho }}_{{\text{f}}} }}{{{{\rho }}_{{{\text{hnf}}}} }}} \right)\left( {\frac{1}{{{\text{Re}}}}} \right)\left( {\frac{{\partial ^{2} }}{{\partial {\text{X}}^{2} }} + \frac{{\partial ^{2} }}{{\partial {\text{Y}}^{2} }}} \right){\text{M}}_{{{\text{p}},{\text{hnf}}}} \\ & \;\;\; - {{\varepsilon }}^{2} {{\delta }}\left( {\frac{1}{{{\text{Re~Da}}}}} \right)\left( {\frac{{{{\rho }}_{{\text{f}}} }}{{{{\rho }}_{{{\text{hnf}}}} }}} \right)\left( {\frac{1}{{\left( {1 - \phi _{{{\text{hnf}}}} } \right)^{{2.5}} }}} \right){{\omega }}_{{{\text{p}},{\text{~hnf}}}} \\ & \;\;\; + {{\varepsilon }}^{2} \left( {\frac{{{\text{Ra}}}}{{{\text{Re}}^{2} {\text{Pr}}}}} \right)\frac{{\left( {{{\rho \beta }}} \right)_{{{\text{hnf}}}} }}{{{{\rho }}_{{{\text{hnf}}}} {{~\beta }}_{{\text{f}}} }}~\frac{\partial }{{\partial {\text{x}}}}\left( {{{\theta }}_{{{\text{p}},{\text{~hnf}}}} } \right) \\ & \;\;\; + {{\varepsilon }}^{2} \frac{{{\text{Ha}}^{2} }}{{{\text{Re}}}}\left( {\frac{{{{\rho }}_{{\text{f}}} }}{{{{\rho }}_{{{\text{hnf}}}} }}} \right)\left( {\frac{{{{\sigma }}_{{{\text{hnf}}}} }}{{{{\sigma }}_{{\text{f}}} }}} \right)\frac{{\partial ^{2} }}{{\partial {\text{x}}^{2} }}\left( {{{\psi }}_{{{\text{p}},{\text{hnf}}}} } \right) \\ \end{aligned}$$

**Angular momentum equation:**

19 $$\begin{gathered} \left( {{\text{U}}_{{{\text{p}},{\text{hnf}}}} \frac{\partial }{{\partial {\text{X}}}} + {\text{V}}_{{{\text{p}},{\text{hnf}}}} \frac{\partial }{{\partial {\text{Y}}}}} \right){\text{M}}_{{{\text{p}},{\text{hnf}}}} = {\upvarepsilon }\left( {\frac{1}{{{\text{Re}}}}} \right)\left( {\frac{{{\uprho }_{{\text{f}}} }}{{{\uprho }_{{{\text{hnf}}}} }}} \right)\left( {\frac{1}{{\left( {1 - \phi_{{{\text{hnf}}}} } \right)^{2.5} }} + \frac{{{\text{K}}_{0} }}{2}} \right)\left( {\frac{{\partial^{2} }}{{\partial {\text{X}}^{2} }} + \frac{{\partial^{2} }}{{\partial {\text{Y}}^{2} }}} \right){\text{M}}_{{{\text{p}},{\text{ hnf}}}} - \left( {\frac{{{\uprho }_{{\text{f}}} }}{{{\uprho }_{{{\text{hnf}}}} }}} \right)\left( {\frac{1}{{{\text{Re}}}}} \right){{ \chi }} \hfill \\ {\text{K}}_{0} \left( {2{\varepsilon M}_{{{\text{p}},{\text{ hnf}}}} - {\upomega }_{{{\text{p}},{\text{ hnf}}}} } \right) \hfill \\ \end{gathered}$$

**Energy equation:**

20 $$\left( {{\text{U}}_{{{\text{p}},{\text{hnf}}}} \frac{\partial }{{\partial {\text{X}}}} + {\text{V}}_{{{\text{p}},{\text{hnf}}}} \frac{\partial }{{\partial {\text{Y}}}}} \right){\uptheta }_{{{\text{p}},{\text{hnf}}}} = \frac{{{\upalpha }^{*} }}{{{\upalpha }_{{\text{f}}} }}\left( {\frac{{\partial^{2} }}{{\partial {\text{X}}^{2} }} + \frac{{\partial^{2} }}{{\partial {\text{Y}}^{2} }}} \right){\uptheta }_{{{\text{p}},{\text{ hnf}}}}$$

21 $${\text{Here,}}\;\;{{\alpha }}_{{{\text{hnf}}}} = \frac{{{\text{k}}_{{{\text{hnf}}}} }}{{\left( {{{\rho C}}_{{\text{p}}} } \right)_{{{\text{hnf}}}} }},{{~~\alpha }}_{{{\text{eff}}}} = \frac{{{\text{k}}_{{{\text{eff}}}} }}{{\left( {{{\rho {\rm C}}}_{{\text{p}}} } \right)_{{{\text{hnf}}}} }},{\text{~k}}_{{{\text{eff}}}} = \left( {1 - {{\varepsilon }}} \right){\text{k}}_{{\text{s}}} + {{\varepsilon k}}_{{{\text{hnf}}}} {\text{~}}$$

The porous layer and clear fluid zone conform to: ${\upvarepsilon } = 1$, ${\updelta } = 0$, ${\upalpha }^{*} = {\upalpha }_{{{\text{hnf}}}}$ in fluid region and ${\upvarepsilon } = {\upvarepsilon }$, ${\updelta } = 1$, ${\upalpha }^{*} = {\upalpha }_{{{\text{eff}}}}$, in porous stratum.

**Dimensionless boundary conditions are:**

The upper (${\Gamma }_{3}$) and lower (${\Gamma }_{6}$) walls have the condition, U = 1; V = M = 0; ${\uptheta }$ = 0 and U = − 1; V = M = 0; ${\uptheta }$ = 1, respectively. Here

22 $$\left. {\begin{array}{*{20}c} {{\Gamma }_{3} = \left\{ {\left( {{\text{X}},{\text{Y}}} \right) \in {\text{R}}^{2} :{\text{Y}} = 1;\;\;{\text{where }}\;0.25 \le {\text{X}} \le 0.75} \right.} \\ {{\Gamma }_{6} = \left\{ {\left( {{\text{X}},{\text{Y}}} \right) \in {\text{R}}^{2} :{\text{Y}} = 0;\;\;{\text{where}}\;{ }0.25 \le {\text{X}} \le 0.75} \right.} \\ \end{array} } \right\}$$

The conditions associated with slanted walls ${\Gamma }_{1}$, ${\Gamma }_{2}$, ${\Gamma }_{4}$, ${\Gamma }_{5}$ are specified as follows: U = V = M = 0; $\frac{{\partial {\theta }}}{\partial n}$ = 0.

23 $$\left.\begin{array}{c}{\Gamma }_{1}=\left\{\left({\text{X}},{\text{Y}}\right)\in {{\text{R}}}^{2}:{\text{Y}}=2\left({\text{X}}-\frac{3}{4}\right);\mathrm{where }~0.75\le {\text{X}}\le 1\right. \\ {\Gamma }_{2}=\left\{\left({\text{X}},{\text{Y}}\right)\in {{\text{R}}}^{2}:{\text{Y}}-\frac{1}{2}=-2({\text{X}}-1);\mathrm{where }~0.75\le {\text{X}}\le 1\right.\\ {\Gamma }_{4}=\left\{\left({\text{X}},{\text{Y}}\right)\in {{\text{R}}}^{2}:{\text{Y}}-1=2\left({\text{X}}-\frac{1}{4}\right);\mathrm{where }~0\le {\text{X}}\le 0.25 \right.\\ {\Gamma }_{5}=\left\{\left({\text{X}},{\text{Y}}\right)\in {{\text{R}}}^{2}:{\text{Y}}-\frac{1}{2}=-2{\text{X}};\mathrm{where }~0\le {\text{X}}\le 0.25\right.\end{array}\right\}$$

The fluid velocities, stresses, temperatures, and temperature differences at the interface of the upper surface of the porous stratum and the fluid layer are considered to be in equilibrium. Assuming the dynamic viscosity of the fluid invariable (${\upmu }_{{\text{p}}} = {\upmu }_{{{\text{hnf}}}}$) in both layers, the interface boundary conditions are defined as:

24 $$\left. {\begin{array}{*{20}c} {{\uppsi }_{{{\text{hnf}}}} = {\uppsi }_{{\text{p}}} ; \frac{{\partial {\uppsi }_{{{\text{hnf}}}} }}{{\partial {\text{X}}}} = \frac{{\partial {\uppsi }_{{\text{p}}} }}{{\partial {\text{X}}}}, {\upomega }_{{{\text{hnf}}}} = {\upomega }_{{\text{p}}} ; \frac{{\partial {\upomega }_{{{\text{hnf}}}} }}{{\partial {\text{X}}}} = \frac{{\partial {\upomega }_{{\text{p}}} }}{{\partial {\text{X}}}}, } \\ {{\uptheta }_{{{\text{hnf}}}} = {\uptheta }_{{\text{p}}} ; \frac{{{\text{k}}_{{{\text{hnf}}}} }}{{{\text{k}}_{{{\text{eff}}}} }}\frac{{\partial {\uptheta }_{{{\text{hnf}}}} }}{{\partial {\text{X}}}} = \frac{{\partial {\uptheta }_{{\text{p}}} }}{{\partial {\text{X}}}}, {\text{ M}}_{{{\text{hnf}}}} = {\text{M}}_{{\text{p}}} ; \frac{{\partial {\text{M}}_{{{\text{hnf}}}} }}{{\partial {\text{X}}}} = \frac{{\partial {\text{M}}_{{\text{p}}} }}{{\partial {\text{X}}}}} \\ \end{array} } \right\}$$

### Nusselt number

The Local Nusselt number at heated bottom wall, which measures the rate of heat transfer inside the hexagonal enclosure, is expressed as:

25 $${\text{Nu}}_{{{\text{loc}}}} = \frac{{{\text{Hq}}_{{\text{w}}} }}{{{\text{k}}_{{\text{f}}} }}$$

here, ${\text{q}}_{{\text{w}}} = - \frac{{{\text{k}}_{{{\text{eff}}}} }}{{\left( {{\text{T}}_{{\text{h}}} - {\text{T}}_{{\text{c}}} } \right)}}\left( {\frac{{\partial {\text{T}}_{{\text{p}}} }}{{\partial {\text{y}}}}} \right)_{{{\Gamma }_{6} }}$ for porous region and ${\text{q}}_{{\text{w}}} = - \frac{{{\text{k}}_{{{\text{hnf}}}} }}{{\left( {{\text{T}}_{{\text{h}}} - {\text{T}}_{{\text{c}}} } \right)}}\left( {\frac{{\partial {\text{T}}_{{{\text{hnf}}}} }}{{\partial {\text{y}}}}} \right)_{{{\Gamma }_{6} }}$ for fluid region.

The “local Nusselt number” is transformed into (26) using dimensionless variables (16).

26 $${\text{Nu}}_{{{\text{loc}}}} = - \frac{{{\text{k}}_{{{\text{eff}}}} }}{{{\text{k}}_{{\text{f}}} }}\left( {\frac{{\partial {\uptheta }_{{\text{p}}} }}{{\partial {\text{Y}}}}} \right)_{{{\Gamma }_{6} }} \;\;{\text{for}}\;{\text{ porous}}\;{\text{ region}}\;{\text{ and}}\;{\text{Nu}}_{{{\text{loc}}}} = - \frac{{{\text{k}}_{{{\text{hnf}}}} }}{{{\text{k}}_{{\text{f}}} }}\left( {\frac{{\partial {\uptheta }_{{{\text{hnf}}}} }}{{\partial {\text{Y}}}}} \right)_{{{\Gamma }_{6} }} \;{\text{for}}\;{\text{ fluid}}\;{\text{ region}}$$

Here, $${\Gamma }_{6} = \left\{ {\left( {{\text{X}},{\text{Y}}} \right) \in {\text{R}}^{2} :{\text{Y}} = 0;{\text{where }}0.25 \le {\text{X}} \le 0.75} \right.$$.

Consequently, the “average Nusselt number (${\text{Nu}}_{{{\text{avg}}}}$)” in non-dimensional form is taken as:

27 $${\text{Nu}}_{{{\text{avg}}}} = \frac{1}{0.5}\mathop \smallint \limits_{0.25}^{0.75} {\text{Nu}}_{{{\text{loc}}}} {\text{dx}}$$

### Entropy generation

Irreversibility analysis refers to the process of measuring the local entropy production resulting from associated fluxes and developed forces. In light of Seyyedi et al.^58^, the dimensionless local entropy production (${{\text{S}}}_{{\text{loc}}}$) for a hybrid nanofluid-saturated porous medium and a micropolar hybrid nanofluid layer in a convective process, influenced by a magnetic field, is expressed as:

28 $$\begin{aligned} {\text{S}}_{{{\text{loc}}}} & = \underbrace {{\frac{{{\text{k}}^{{\text{*}}} }}{{{\text{k}}_{{\text{f}}} }}\left[ {\left( {\frac{{\partial {{\theta }}}}{{\partial {\text{X}}}}} \right)^{2} + \left( {\frac{{\partial {{\theta }}}}{{\partial {\text{Y}}}}} \right)^{2} } \right]}}_{{\begin{array}{*{20}c} {Entropy~Generation~due~} \\ {heat~transfer~\left( {{\text{S}}_{{{\text{HT}}}} } \right)} \\ \end{array} }} \\ & \;\;\; + \underbrace {{{{\xi }}\left( {\frac{{{{\mu }}_{{{\text{hnf}}}} }}{{{{\mu }}_{{\text{f}}} }} + {\text{K}}_{0} } \right)\left[ {\frac{{{\delta }}}{{{\text{Da}}}}\left( {{\text{U}}^{2} + {\text{V}}^{2} } \right) + \left( {2\left( {\frac{{\partial {\text{U}}}}{{\partial {\text{X}}}}} \right)^{2} + 2\left( {\frac{{\partial {\text{V}}}}{{\partial {\text{Y}}}}} \right)^{2} + \left( {\frac{{\partial {\text{U}}}}{{\partial {\text{Y}}}} + \frac{{\partial {\text{V}}}}{{\partial {\text{X}}}}} \right)^{2} } \right)} \right]}}_{{{\text{Entropy~Generation~due~to~fluid~friction~}}\left( {{\text{S}}_{{{\text{FF}}}} } \right)}} \\ & \;\;\; + \underbrace {{\left( {\frac{{{{\sigma }}_{{{\text{hnf}}}} }}{{{{\sigma }}_{{\text{f}}} }}} \right){{\xi {\text Ha}}}^{2} {\text{V}}^{2} }}_{{{\text{Entropy~Generation~due~to~magnetic~forces~}}\left( {{\text{S}}_{{{\text{MF}}}} } \right)}} \\ \end{aligned}$$

Here, ${\text{k}}^{*} = {\text{k}}_{{{\text{eff}}}} ;{\updelta } = 1;{\uptheta } = {\uptheta }_{{\text{p}}} ; {\text{U}} = {\text{U}}_{{\text{p}}} ; {\text{V}} = {\text{V}}_{{\text{p}}}$ for porous medium and ${\text{k}}^{*} = {\text{k}}_{{{\text{hnf}}}} ;{\updelta } = 0;{{ \uptheta }} = {\uptheta }_{{{\text{hnf}}}} ; {\text{U}} = {\text{U}}_{{{\text{hnf}}}} ; {\text{V}} = {\text{V}}_{{{\text{hnf}}}}$ for clear fluid region. Also, ‘${\upxi } = \frac{{{\upmu }_{{\text{f}}} {\text{T}}_{0} }}{{{\text{k}}_{{\text{f}}} }}\left( {\frac{{{\upalpha }_{{\text{f}}}^{2} }}{{{\text{H}}\left( {{\Delta T}} \right)^{2} }}} \right)$’ denotes the irreversible distribution. The estimated value of the total entropy generation (${\text{S}}_{{{\text{Total}}}}^{ }$) can be obtained by:

29 $${\text{S}}_{{{\text{Total}}}}^{ } = \mathop \smallint \limits_{{\text{V}}}^{ } {\text{S}}_{{{\text{loc}}}} {\text{ dV}}$$

The “local (${\text{Be}}_{{{\text{local}}}}$) and average (${\text{Be}}_{{{\text{avg}}}}$) Bejan number,” can be defined as:

30 $${\text{Be}}_{{{\text{local}}}} { } = \frac{{{\text{S}}_{{{\text{HT}}}} }}{{{\text{S}}_{{{\text{loc}}}} }}\;\;{\text{and}}\;\;{\text{Be}}_{{{\text{avg}}}} = \mathop \smallint \limits_{{\text{V}}}^{ } {\text{Be}}_{{{\text{local}}}} {\text{ dV}}$$

## Numerical solution methodology

**Figure 2:**
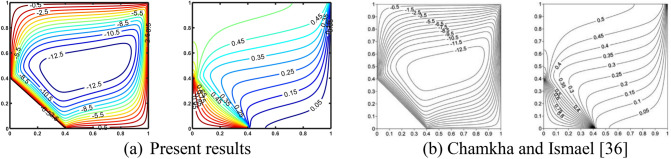
Validation of self-created codes is performed through the assessment of isotherms and streamlines at Ra = 800; Kr = 1; D = 0.4 with the work of Chamkha and Ismael ^36^.

**Figure 3:**
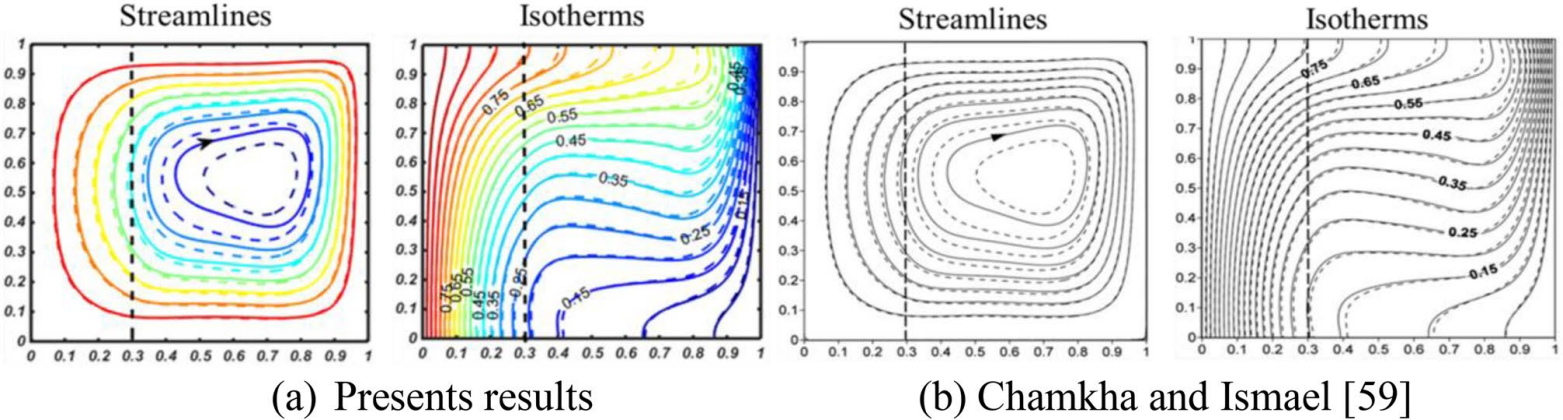
Comparison of isotherms and streamlines as a means of validating independently developed code together with particular outcome of Chamkha and Ismael^59^ when “Ra = 10^5^, Pr = 6.26,${\text{ X}}_{{\text{p}}} = 0.3,$ Da = 10^–5^,${ }\phi_{{{\text{cu}}}} = 0{ }\left( {\text{solid lines}} \right)$, and $\phi_{{{\text{cu}}}} = 0.05{ }\left( {\text{dashed lines}} \right)$ for ${\text{A}}=1$”.

To solve non-dimensional “governing Eqs. (17–20)” numerically, together with dimensionless boundary conditions given above in Eqs. (22–24), the “Finite Difference Method” associated with “successive over relaxation (SOR) and successive under relaxation (SUR)” is employed. Self—developed MATLAB codes are used to compute the desired results. Validation of self-created codes is performed through the assessment of isotherms and streamlines with the works of Chamkha and Ismael^36,59^, as displayed in Figs. 2 and 3. The excellent correlation between the outcomes confirms the validity of our simulation. The grid independence of the self-developed MATLAB codes was examined by computing the “average Nusselt number (${\text{Nu}}_{{{\text{avg}}}}$) and average Bejan number (Be_avg_)” at the bottom heated wall, as displayed in Table 3. Therefore, in order to attain the intended results, the 241 × 121 grids were used.

**Table 3:** Grid independency test when Re = 25, Ra = 10^5^, Da = 10^–5^, Ha = 10, K_0_ = 2.0, and $4\%$, X_p_ = 0.5.

Grid size	161 × 81	181 × 91	201 × 101	221 × 111	241 × 121
${\text{Nu}}_{{{\text{avg}}}}$	5.4546	5.2512	5.1548	5.1338	5.1329
${\text{Be}}_{{{\text{avg}}}}$	0.8555	0.7842	0.7298	0.7092	0.7085

## Results and discussion

Throughout this article, we will explore novel findings regarding the movement of a fluid confined within a partially porous enclosure with a hexagonal-shaped cross-section. It should be noted that the slanted sides of the hexagonal enclosure are thermally insulated; however, the top and bottom walls are kept at temperatures T_c_ and T_h_ respectively, i.e., T_h_ $>$ T_c_. Additionally, the top and bottom walls move at a constant velocity ‘U_0_’ but in opposite directions, as depicted in Fig. 1. A micropolar hybrid nanofluid-saturated porous layer having a thickness ‘X_p_ = 0.5’ is positioned on the left side of the enclosure. However, the rest of the enclosure filled with a clear micropolar hybrid nanofluid. The boundary between the porous and clear fluid regions is considered to be permeable, allowing for the possibility of cross-flow across both regions. This investigation employs the Ag‐MgO/water-based hybrid nanofluid. The numerical outcomes are demonstrated through graphical representation, demonstrating the impact of the analyzed variables on flow, heat exchange, and irreversibilities. The thermal conductivity of the porous material is ${\text{k}}_{{\text{s}}}$ = 0.845 W mK^−1^, and its porosity is kept at ε = 0.398, making it identical to 3 mm glass beads. The investigated parameters have the following ranges: Ra = 10^4^–10^6^, Da = 10^–3^–10^–5^, Ha = 0–20, K_0_ = 2.0–8.0; Re = 10–50; X_p_ = 0.3–0.7; $\phi_{{{\text{hnf}}}}$ = 0–4%.

**Figure 4:**
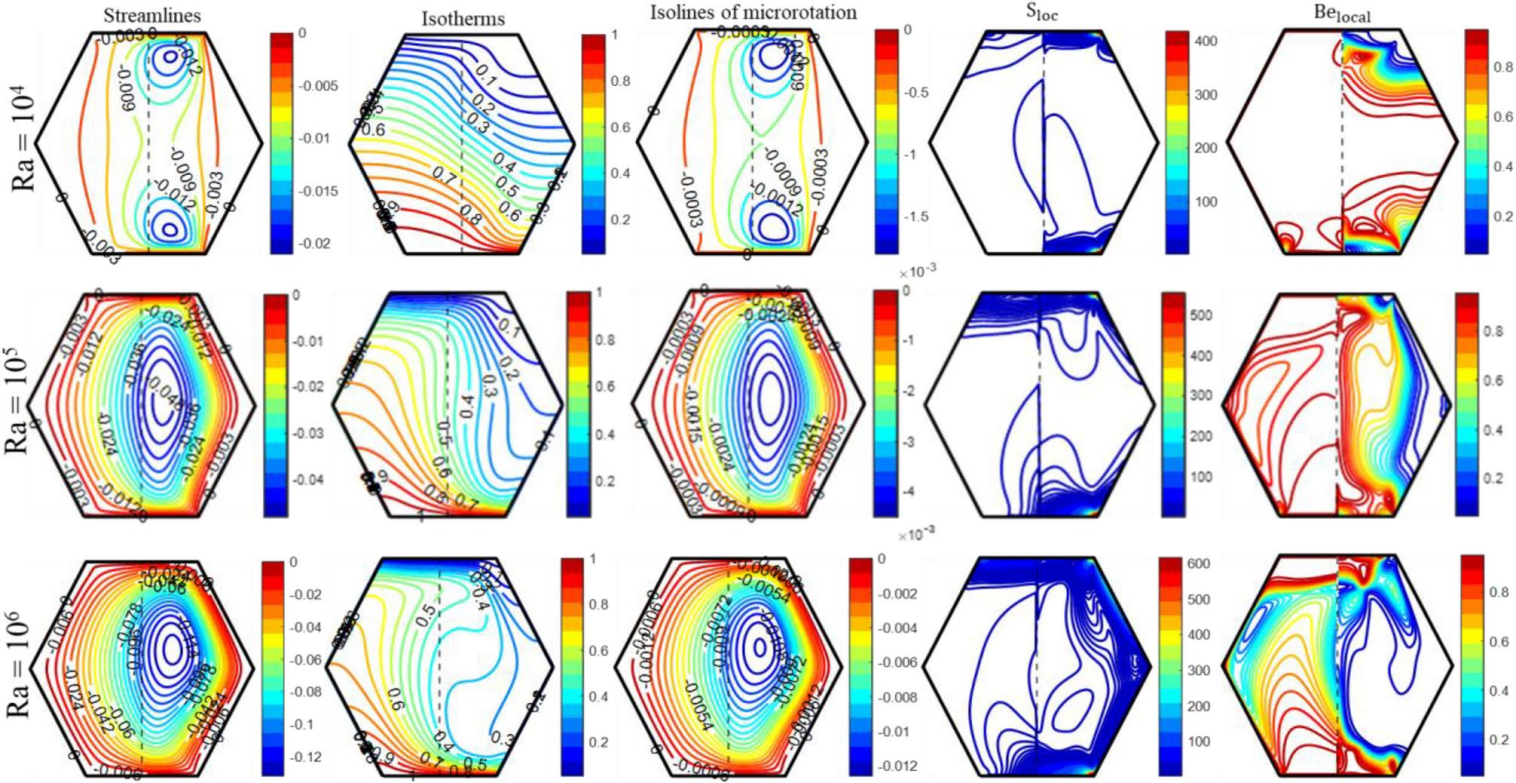
The impact of ‘Ra’ on the patterns of “streamlines (${\uppsi }$), isotherms (${\uptheta }$), isolines of microrotation (${\text{N}}$), S_loc_, and Be_local_” when Da = 10^–5^, Ha = 10, K_0_ = 2.0; Re = 25; $\phi_{{{\text{hnf}}}}$ = 4%; X_p_ = 0.5.

The significance of buoyancy forces on flow circulation, transmission of heat, and production of entropy is illustrated in Fig. 4. In light of the dominance of inertial forces over buoyancy forces at a low ‘Ra’ value, i.e., Ra = 10^4^, two circulating cells emerge in close proximity to the moving boundaries of the enclosure. The enlargement of these cell circulations is towards the right side of the enclosure as a result of the existence of a porous medium on the left side, which limits the movement of fluid in this region. An elevation in ‘Ra’ from 10^4^ to 10^6^ corresponds to an augmentation in the buoyancy force over inertial forces, which in turn leads to the intensification of circulation in both the fluid and porous regions. Additionally, both circulating cells merge to form a single vortex cell as the ‘Ra’ ascends because the fluid in close proximity to the substantially heated bottom side of the cavity is considerably hotter in comparison to the fluid near the cold top wall and moves in the opposite x-axis direction as a result of the wall’s movement in that direction, whereas the fluid near the cold top wall moves in the positive x-direction. This results in bidirectional fluid flow, with the fluid moving both upwards and downwards along the inclined cold walls. It is observable that as the Ra value increases from 10^4^ to 10^6^, the circulating cells become enlarged and strengthened. Furthermore, as Ra increases from 10^4^ to 10^6^, ${\uppsi }_{{{\text{max}}}}$ goes from 0.04 to 0.15. The influence of ‘Ra’ on the mechanism of heat convection is depicted by the contours of isotherms. The fact that isotherms are almost straight lines at Ra = 10^4^ suggests that heat is conveyed via conduction at a low ‘Ra’ value. Although, as ‘Ra’ grows, the curvature of the isotherms changes drastically in both regions. Compared to the fluid region, the porous region exhibits a significantly larger hot region, which leads to a higher retention of heat within it. As a result, the porous region experiences comparatively less heat convection than the fluid region. An increase in the Rayleigh number (Ra) results in a decrease in the thickness of the boundary layer, which in turn enhances heat convection. Moreover, the isolines of micro-rotation exhibit an identical pattern to that of streamlines, although the magnitudes are different. Meanwhile, the effect of buoyancy forces on ‘S_loc_’ and ‘Be_local_’ has been depicted in Fig. 4. Three factors—magnetic field, heat transfer, and fluid friction, contribute to entropy production. Figure 4 clearly illustrates that entropy production exclusively takes place in the vicinity of the moving wall within the fluid region, specifically for low 'Ra' values. As the value of Ra increases, the generation of entropy intensifies. Moreover, Fig. 4 demonstrates the ‘Be_local_’ behavior for different ‘Ra’ values. The Be_local_ is determined by “the ratio of the local heat transfer irreversibility to the total local irreversibility resulting from the transfer of heat, fluid friction, and magnetic forces.” Regions characterized by significant temperature fluctuations and low velocity magnitudes exhibit the highest values of Be_local_, while areas with stable temperatures and high velocity magnitudes exhibit its lowest values. Therefore, the Be_avg_ value is greater in the porous region than in the fluid region. Therefore, thermal irreversibilities have a greater influence than frictional irreversibilities in the porous region when Ra = 10^4^. At higher values of Ra, the velocity of the fluid grows, which leads to the dominance of frictional irreversibility over thermal irreversibility.

**Figure 5:**
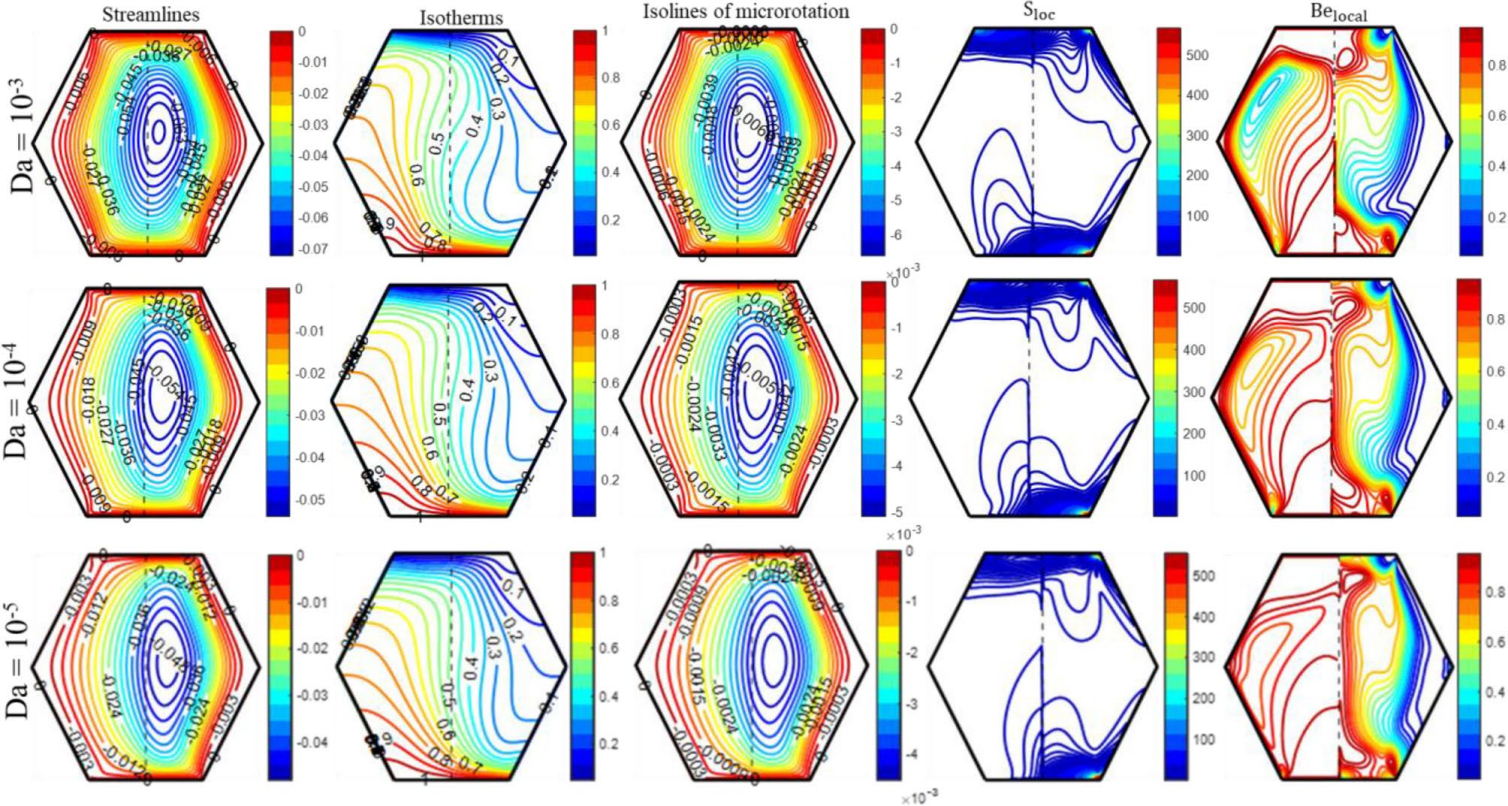
The impact of 'Da' on the patterns of “streamlines (${\uppsi }$); isotherms (${\uptheta }$); isolines of microrotation (${\text{N}}$); S_loc_; and Be_local_” when Ra = 10^5^, Ha = 10, K_0_ = 2.0; Re = 25; $\phi_{{{\text{hnf}}}}$ = 4%; X_p_ = 0.5.

Figure 5 displays the influence of porous media on the “streamlines, isotherms, isolines of micro-rotation, local entropy generation, and local Bejan number,” precisely associated with the Darcy number (Da). A circulation cell forms within the enclosure for each ‘Da’ value, as the upper wall moves in the positive x-axis direction and the bottom wall moves in the opposite direction. This circulating cell strengthens the porous layer via a clear fluid zone. A decrease in ‘Da’ from 10^–3^, to 10^–5^ causes a reduction in permeability, leading to a decrease in the intensity and size of the cell circulation and an increase in resistance to fluid flow within the porous region. The isotherms close to the moving bottom wall are more compact for Da = 10^–3^ in contrast to the findings obtained for Da = 10^–4^ and 10^–5^. Diminishing the Darcy number reduces the permeability of the medium, resulting in increased heat retention in the fluid. This leads to an upsurge in the fluid’s temperature within the porous layer for lower Darcy values, ultimately reducing heat convection.

**Figure 6:**
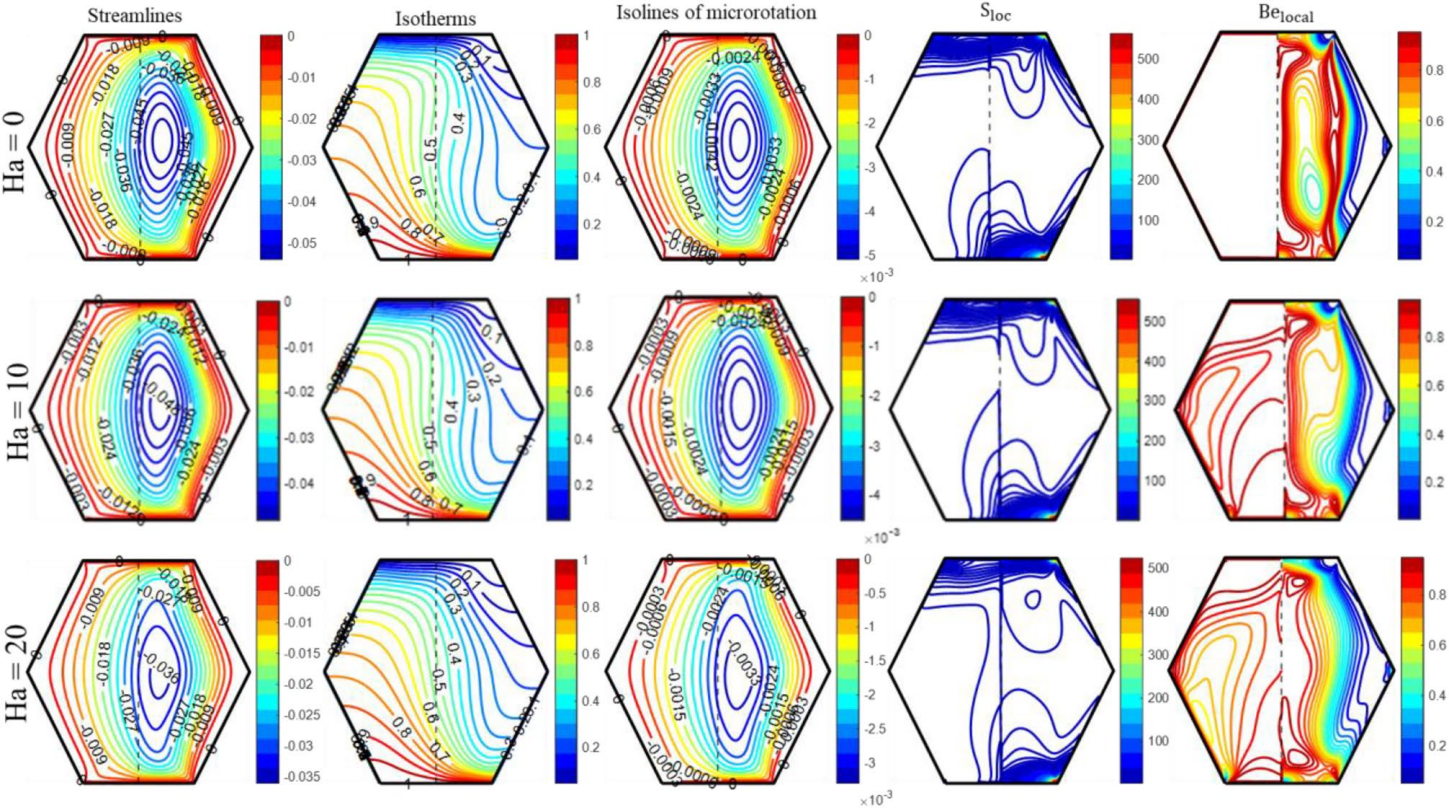
The impact of 'Ha' on the patterns of “streamlines (${\uppsi }$); isotherms (${\uptheta }$); isolines of microrotation (${\text{N}}$); S_loc_; and Be_local_” when Ra = 10^5^, Da = 10^–5^, K_0_ = 2.0; Re = 25; $\phi_{{{\text{hnf}}}}$ = 4%; X_p_ = 0.5.

A boost in Darcy’s number results in the bending of the isotherms in the pure fluid region, which is correlated with an improvement in heat convection. The diminution in heat convection is more pronounced in the porous region compared to the clear fluid region, as the Darcy number decreases from 10^–3^ to 10^–5^, as shown in Fig. 11. As ‘Da’ drops from 10^–3^ to 10^–5^, the data in Table 4 shows that Nu_avg_ drops by 23.52%. Meanwhile, the adverse effect of ‘Da’ on horizontal and vertical velocity distributions is illustrated in Fig. 11. Furthermore, Fig. 5 illustrates a drop in the intensity and magnitude of micro-rotation isolines as Da reduces from 10^–3^ to 10^–5^. Additionally, the S_loc_ intensifies along the lower and upper walls of the enclosure, as depicted in Fig. 5. The ‘S_loc_’ reaches its maximum value in the lower right corners of the enclosure and drops as the ‘Da’ value diminishes. The permeability of the porous medium significantly affects the ‘Be_local_’. An observation has been made that decreasing the value of ‘Da’ from 10^–3^ to 10^–5^ results in an increase in the region where thermal irreversibility is dominant. Figure 6 demonstrates the consequences of Hartmann number (Ha) on “streamlines, isotherms, isolines of micro-rotation, local entropy generation, and local Bejan number.” As shown in Fig. 6, the results of streamline contours clearly show that the strength of flow circulation decreases as the ‘Ha’ value increases. Furthermore, the fluid's velocity decreases significantly as ‘Ha’ advances. The most probable cause for this is that the presence of a magnetic field generates Lorentz force, which reduces the fluid’s motion. Meanwhile, the magnetic field has a discernible effect on the core cells, whereas the cells near the boundary edges show no significant changes.

**Table 4:** Influence of different parameters on Nu_avg_, S_Total_ and Be_avg_.

Ra	Da	Ha	K_0_	Re	$\phi_{{{\text{hnf}}}}$	X_p_	Nu_avg_	S_Total_	Be_avg_
10^5^	10^–5^	10	2.0	25	0.04	0.5	5.1329	6.2695	0.7085
10^4^	–	–	–	–	–	–	2.1622	3.6912	0.9901
10^6^	–	–	–	–	–	–	7.4589	14.247	0.4581
10^5^	10^–3^	–	–	–	–	–	6.7115	8.0135	0.7357
–	10^–4^	–	–	–	–	–	5.7681	6.8837	0.7262
–	10^–5^	0	–	–	–	–	5.3750	6.3637	0.7335
–	–	20	-	-	-	-	4.3940	5.8231	0.6525
–	–	10	5.0	–	–	–	4.3889	6.8326	0.5557
–	–	–	8.0	–	–	–	3.8367	7.6692	0.4367
–	–	–	2.0	10	–	–	3.4036	6.2979	0.4976
–	–	–	–	50	–	–	7.9964	8.1237	0.8043
–	–	–	–	25	0.02	–	4.9828	6.1827	0.7090
–	–	–	–	–	0.00	–	4.8287	6.0939	0.7092
–	–	–	–	–	0.04	0.3	7.1224	8.7544	0.6846
–	–	–	–	–	–	0.7	1.3965	2.3870	0.7989

**Figure 7:**
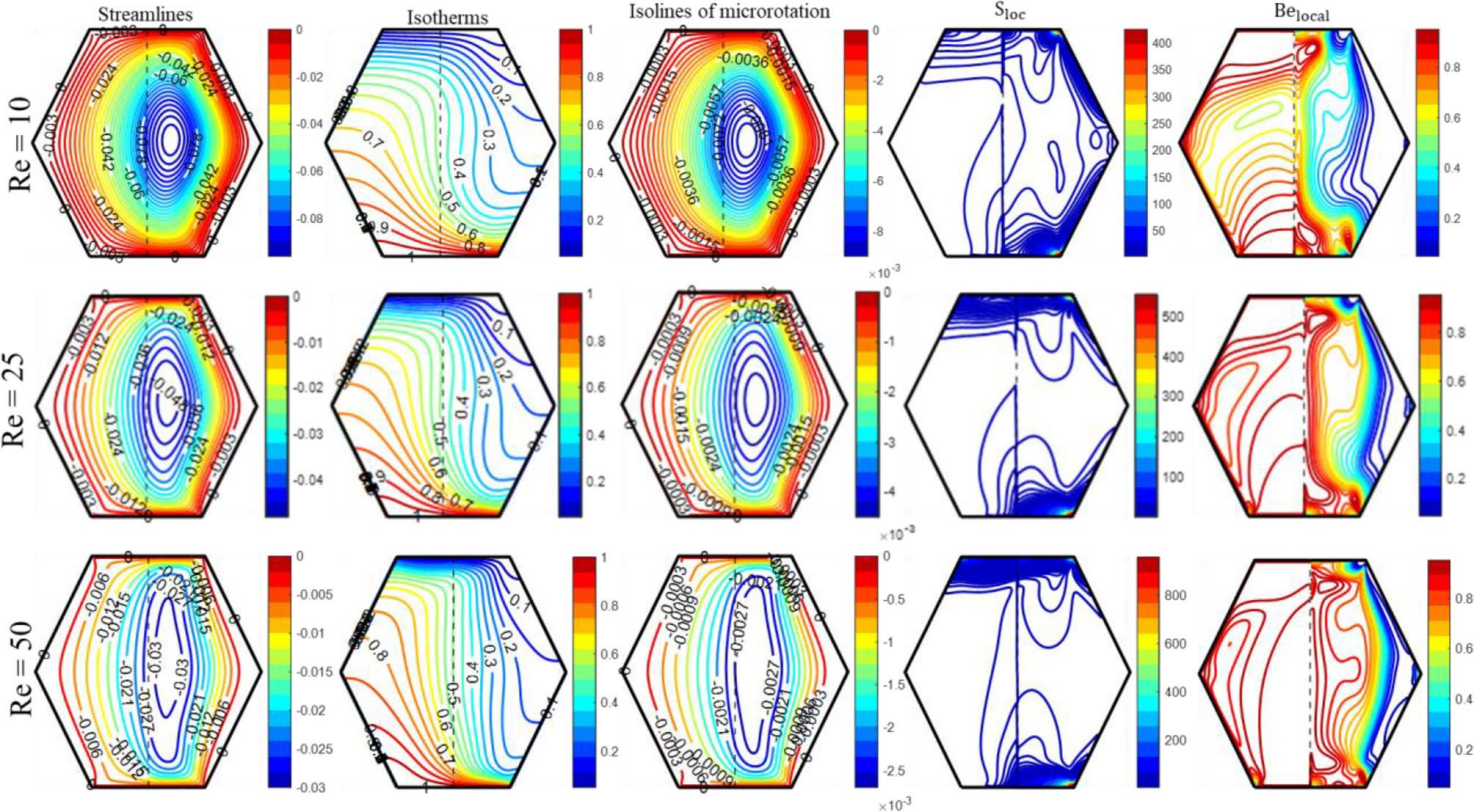
The impact of 'Re' on the patterns of “streamlines (${\uppsi }$); isotherms (${\uptheta }$); isolines of microrotation (${\text{N}}$); S_loc_; and Be_local_” when Ra = 10^5^, Da = 10^–5^, K_0_ = 2.0; Ha = 10; $\phi_{{{\text{hnf}}}}$ = 4%; X_p_ = 0.5.

Additionally, Fig. 6 demonstrates the impact of ‘Ha’ on isotherm contours within the enclosure. As the Hartmann number grows, the thickness of the thermal boundary layer also grows, resulting in a drop in heat convection from the bottom heated wall. Since the isotherm lines in the fluid layer exhibit more curvature than those in the porous layer for each value of ‘Ha’, a greater amount of heat is transferred across the fluid layer. This observation is backed up by the “local Nusselt number (Nu_loc_)” profiles provided in Fig. 11. “Isolines of micro-rotation” weaken and become less dense as ‘Ha’ improves. The presence of substantial temperature variations and low fluid velocities in the porous layer, in contrast to the fluid layer, suggests that heat transfer mainly depends on temperature differences instead of the movement of fluid in the porous layer. Therefore, thermal irreversibility are more prominent compared to frictional irreversibility in porous layers. However, frictional irreversibility’s takes place in a fluid layer because of the substantial fluid flow and shear stress. Entropy is predominantly generated in areas characterized by significant disparities in temperature or velocity; thus, the majority of entropy is produced in close proximity to the top and bottom walls of the enclosure when Ha = 0. Consequently, when ‘Ha’ increases from 0 to 20, the fluid’s velocity decreases as a result of the appearance of Lorentz forces, which causes a reduction in entropy production. Figure 7 depicts the impact of Reynolds number (Re) on the contours of “flow, thermal, and local irreversibility’s.” The top and bottom walls move in opposite directions, with the upper wall moving to the right and the bottom one moving to the left. Furthermore, an escalation in the magnitude of ‘${\text{Re}}$’ serves as a measure of the velocity of the walls.

Figure 7 shows that the influence of shear effects diminishes at low Re = 10, and buoyancy forces entirely govern the circulation. This leads to the formation of a circular flow pattern within the enclosure due to the opposing movements of the top and bottom walls. In contrast to the porous layer, the fluid layer has denser streamlines, allowing the fluid to move more quickly through it. At Re = 50, the motion of the lid produces a more substantial shear effect compared to the buoyant force, resulting in the formation of a circulating cell that stretches towards both the top and bottom walls of the hexagonal enclosure. The isotherm contours demonstrate that as the wall’s velocity increases, the thermal boundary layer decreases, resulting in enhanced heat transfer through this process. The fact that the hot zone grows in the porous area as Re rises suggests that the porous layer transfers less heat. The movement of walls has a similar effect on the isolines of micro-rotation to streamlines, but with varying intensity. Furthermore, when the Reynolds number is low, there is a greater generation of entropy in the fluidic region along the right slanted walls of the enclosure. However, as ‘Re’ increases from 10 to 50, the production of entropy is solely attributed to the motion of the enclosure walls. There are thermal irreversibilities in the porous zone and frictional irreversibilities in the fluidic zone at low Re values. An increase in Re results in a rise in the hot zone, which indicates the presence of significant temperature differences and low flow velocity in the porous region, leading to the prevalence of thermal irreversibilities. In the context of microfluidics, the circulation of fluid is caused by the translation and rotation of granules. Feng et al. ^60^ examined the fact that the antisymmetric part of the deviatoric stress accounts for the motion of the solution at the suspension scale. The vortex viscosity “κ” gives rise to a non-dimensional factor called the “vortex viscosity parameter (K_0_).” ‘K_0_’ is a physical parameter that is employed to analyze the non-Newtonian rheology of fluids. K_0_ = 0, for a Newtonian fluid.

**Figure 8:**
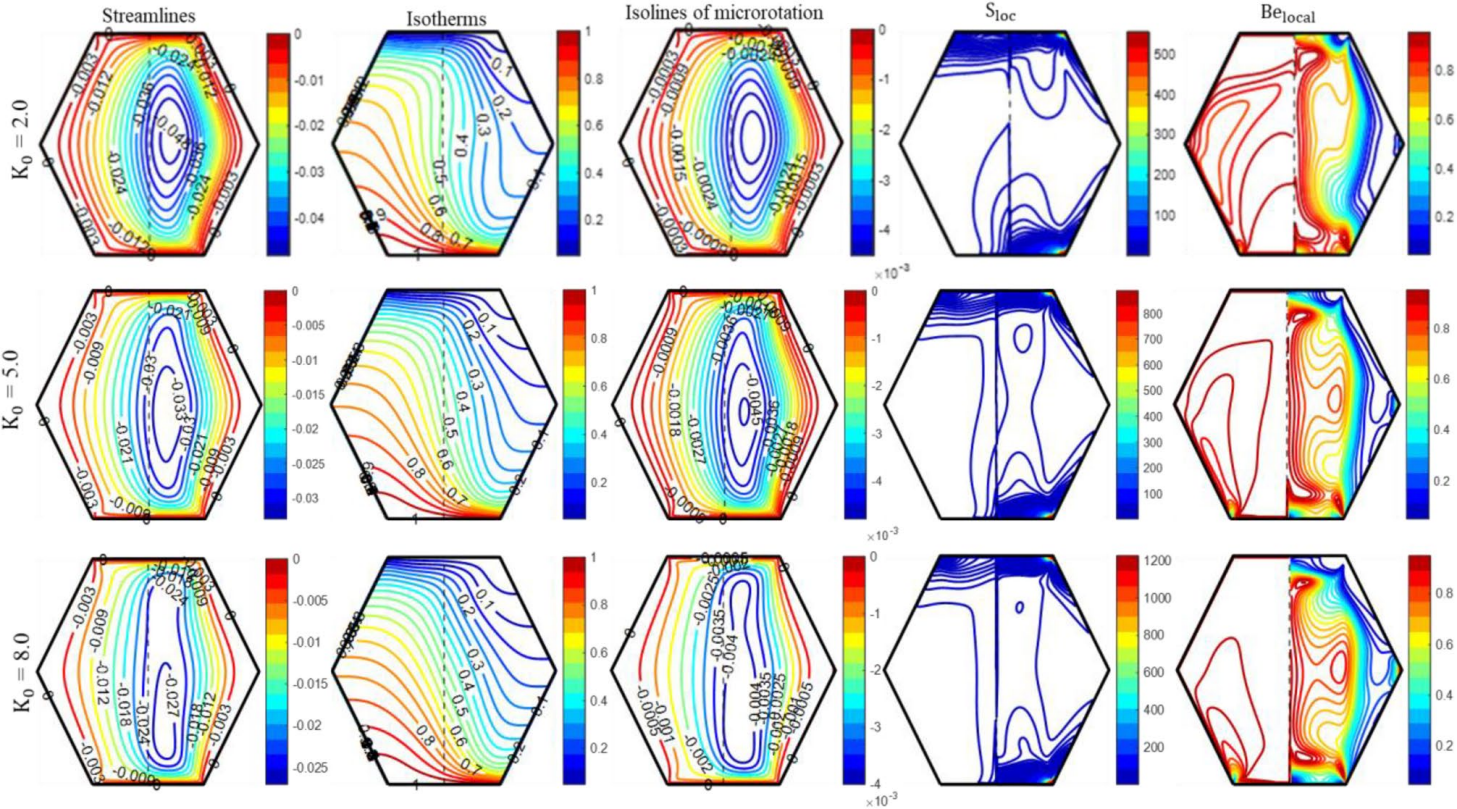
The impact of ‘K_0_’ on the patterns of “streamlines (${\uppsi }$); isotherms (${\uptheta }$); isolines of microrotation (${\text{N}}$); S_loc_; and Be_local_” when Ra = 10^5^, Da = 10^–5^, Re = 25; Ha = 10; $\phi_{{{\text{hnf}}}}$ = 4%; X_p_ = 0.5.

**Figure 9:**
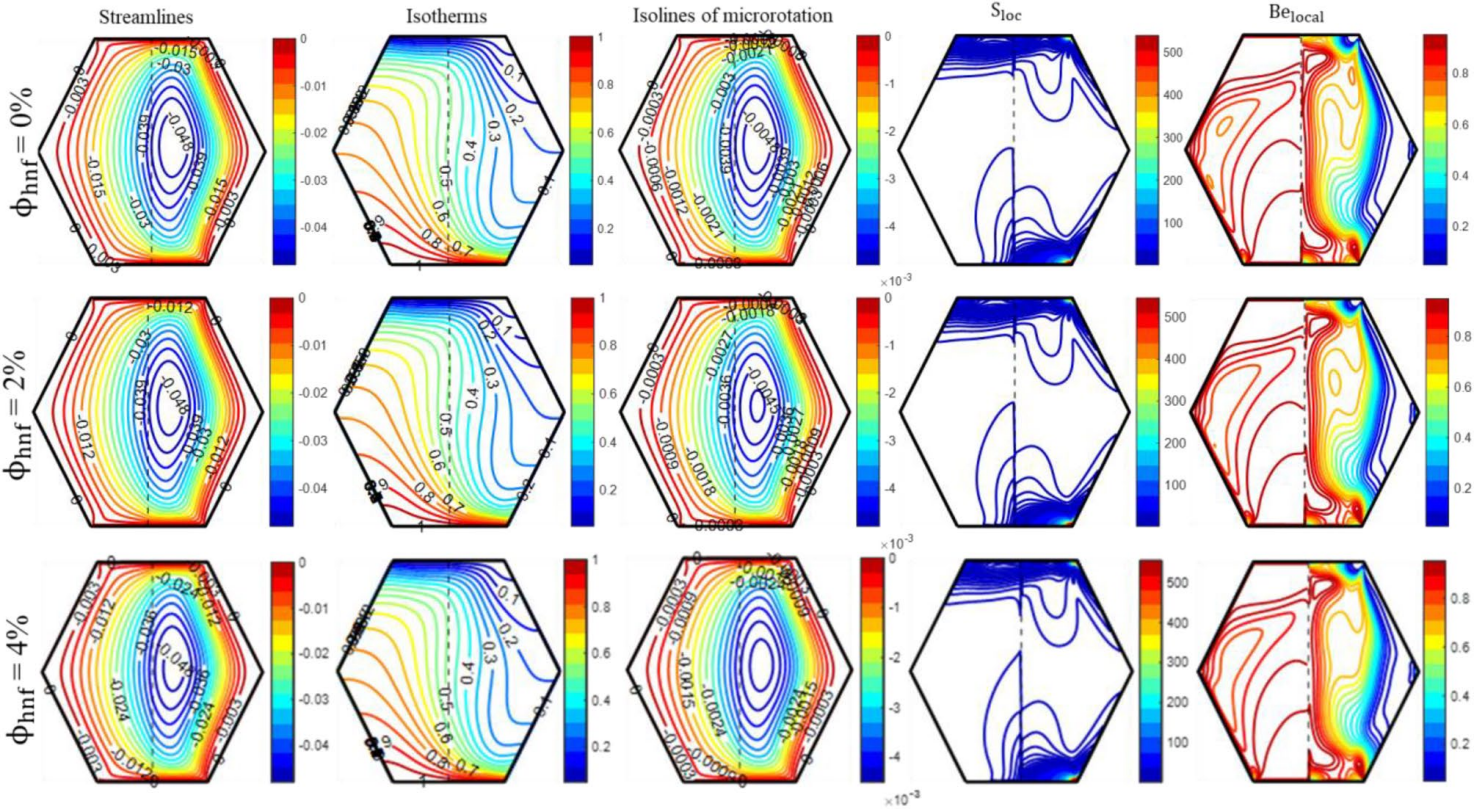
The impact of ‘$\phi_{{{\text{hnf}}}}$’ on the patterns of “streamlines (${\uppsi }$); isotherms (${\uptheta }$); isolines of microrotation (${\text{N}}$); S_loc_; and Be_local_” when Ra = 10^5^, Da = 10^–5^, Re = 25; Ha = 10; K_0_ = 2.0; X_p_ = 0.5.

Figure 8 demonstrates the impact of non-Newtonian rheology on the patterns of “streamlines, isotherms, isolines of micro rotation, S_loc_, and Be_local_.” A boost in the value of ‘K_0_’ leads to a notable reduction in the occurrence of streamline bunching for both regions. A decline in |${\uppsi }_{{{\text{max}}}} |$ from 0.048 to 0.027 occurs when ‘K_0_’ increases from 2 to 8. The outcomes accord with the previous investigations showing that both the fluid’s velocity and dynamic viscosity reduce with a boost in K_0_. The isothermal lines straighten out as ‘K_0_’ rises. The isotherm lines near the heated bottom wall are denser for K_0_ = 2, compared to K_0_ = 8. Consequently, this results in an enlargement of the region with high temperatures inside the enclosure, particularly in the porous region. An escalation in K_0_ leads to an increase in resistance to fluid movement, which in turn reduces heat transfer. Furthermore, Fig. 8 demonstrates a decrease in both the size and strength of the “isolines of micro-rotation” with a boost in ‘K_0_’. Finally, the effects of the fluid’s rheology on the production of entropy through the elevation of ‘K_0_’ are illustrated in Fig. 8. As K_0_ increases, entropy production also increases. Furthermore, an increase in K_0_ corresponds to an increase in the vicinity of high thermal gradients and low velocity, indicating that thermal irreversibilities have a greater influence than frictional irreversibilities, particularly in porous layer. Although close to right-slanted walls, the velocity is relatively high. As a result, frictional irreversibility becomes more significant than thermal irreversibility. Figure 9 explains the effects of $\phi_{{{\text{hnf}}}}$ in the fluid. An upsurge in $\phi_{{{\text{hnf}}}}$ significantly affects the distribution of cell circulation near the center of the circular eddy, but no substantial modifications appear near the exterior cells. The streamlines exhibit greater density within the fluid region as opposed to the porous region, indicating a higher velocity within the fluid region. The density of the streamlines diminishes as the value of $\phi_{{{\text{hnf}}}}$ advances. The presence of nanoparticles induces a flow barrier that reduces fluid motion.

Figure 9 demonstrates that $\phi_{{{\text{hnf}}}}$ has a minimal impact on the appearance of isotherm fluctuation. Furthermore, the addition of nanoparticles to a standard fluid increases the thermal conductivity of the fluid. Furthermore, the curvature of the isotherms decreases as the $\phi_{{{\text{hnf}}}}$ increases from 0 to 4%. Additionally, the thermal lines become more concentrated near the heated walls, indicating an increase in heat convection. Figure 9 illustrates a significant influence of $\phi_{{{\text{hnf}}}}$ on the characteristics of micro-rotation isolines, which are identical to streamlines but fluctuate in magnitude. The local entropy production rate within the enclosure increases as the magnitude of $\phi_{{{\text{hnf}}}}$ rises. Because of the substantial temperature fluctuation and reduced speed within the porous region, the maximum value of the Bejan number occurs in the porous region, indicating that thermal irreversibilities are more significant than frictional irreversibilities. In contrast, frictional irreversibilities play a significant role in the fluid layer.

**Figure 10:**
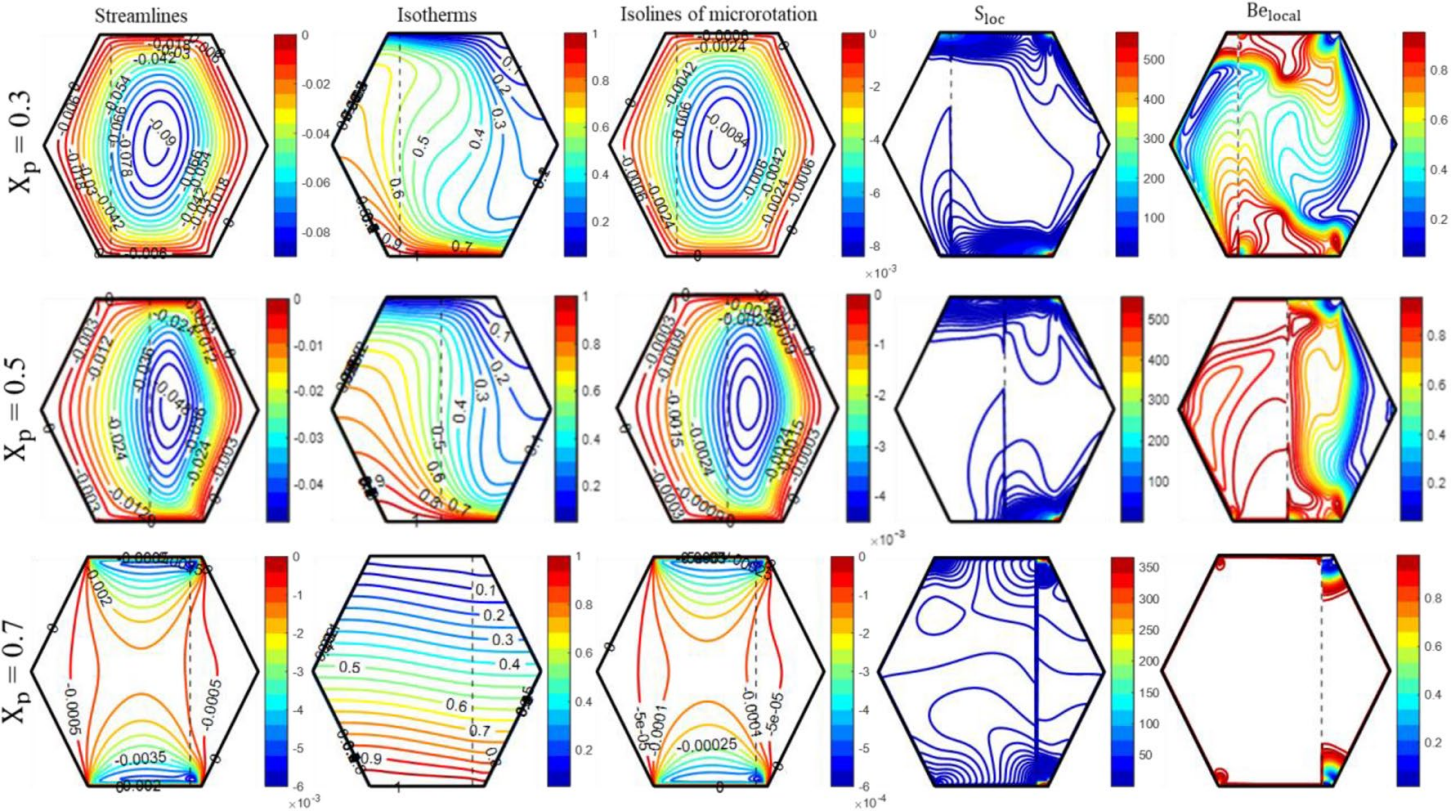
The impact of ‘X_p_’ on the patterns of “streamlines (${\uppsi }$); isotherms (${\uptheta }$); isolines of microrotation (${\text{N}}$); S_loc_; and Be_local_” when Ra = 10^5^, Da = 10^–5^, Re = 25; Ha = 10; K_0_ = 2.0; $\phi_{{{\text{hnf}}}}$ = 4%.

The influence of the thickness of the porous layer (X_p_) on the distribution of streamlines, isotherms, S_loc_, and Be_local_ can be observed in Fig. 10. At the minimum thickness of the porous layer (X_p_ = 0.3), the resistance caused by the porous material is quite small and has low viscous forces. As a result, there is an increased flow throughout the whole enclosure, particularly within the fluid layer. The drag force exerted by porous medium rises proportionally with its thickness. As a result, two circulation cells form at X_p_ = 0.7, restricting fluid movement to the vicinity of the moving walls. Additionally, ‘X_p_’ exerts a substantial influence on the isotherm curves. At X_p_ = 0.3, the isotherms are thicker in the vicinity of the heated wall and display pronounced curvature, resulting in enhanced heat transfer compared to X_p_ = 0.7. The increase in the thickness of the porous layer led to a decrease in entropy production. At X_p_ = 0.3, the presence of thermal irreversibilities is more noticeable in close proximity to the moving walls as a result of the high temperature gradient, while frictional irreversibilities are more prominent in the remaining parts of the enclosure. As the thickness of the porous layer increases from 0.3 to 0.7, there is a decrease in both heat transfer and velocity. This causes the hot zone within the enclosure to rise, resulting in an increase in thermal irreversibilities throughout the enclosure.

**Figure 11:**
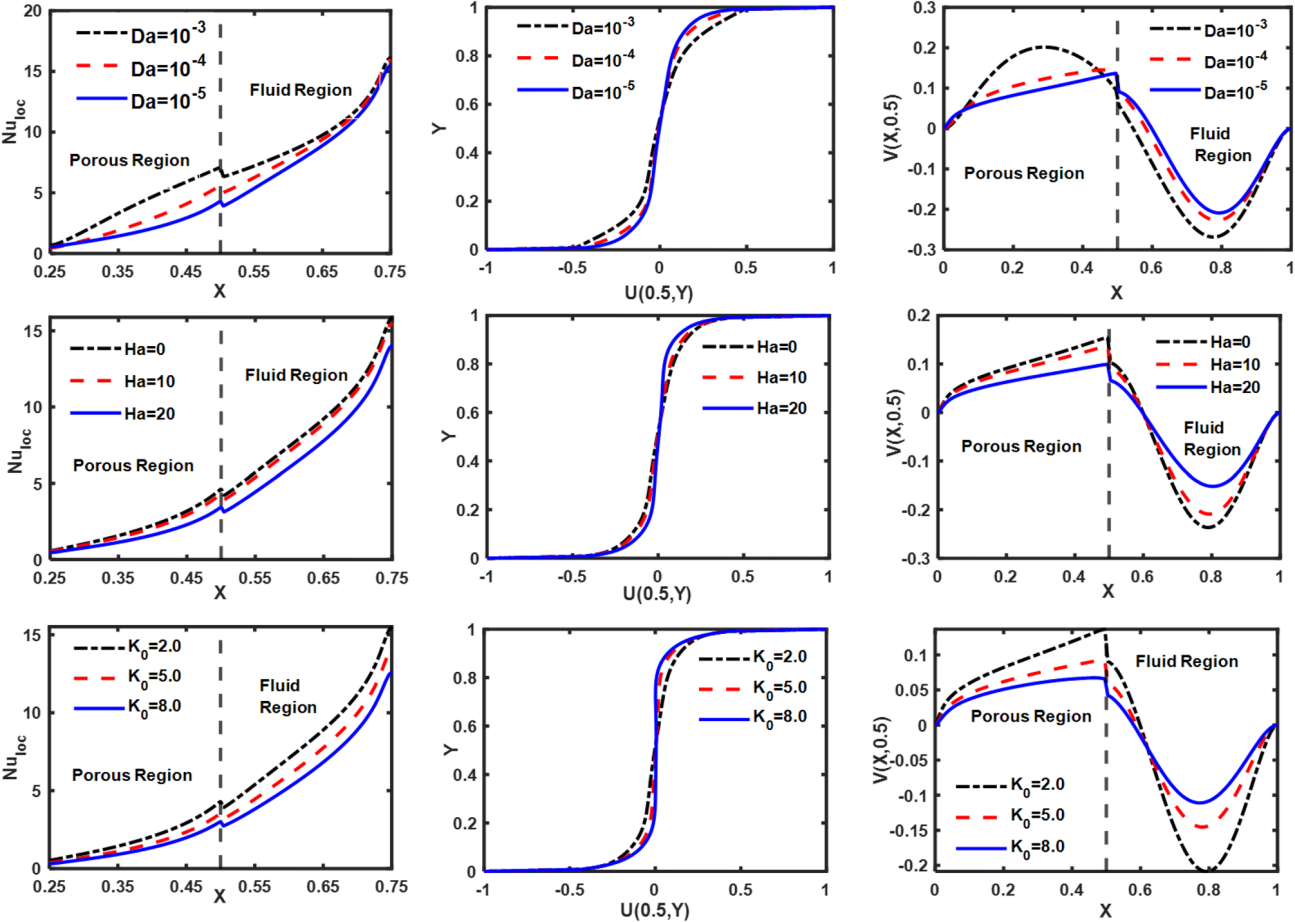
Influence of ‘Da’, ‘Ha’ and ‘K_0_’ on velocity and Nu_loc_ profiles.

**Figure 12:**
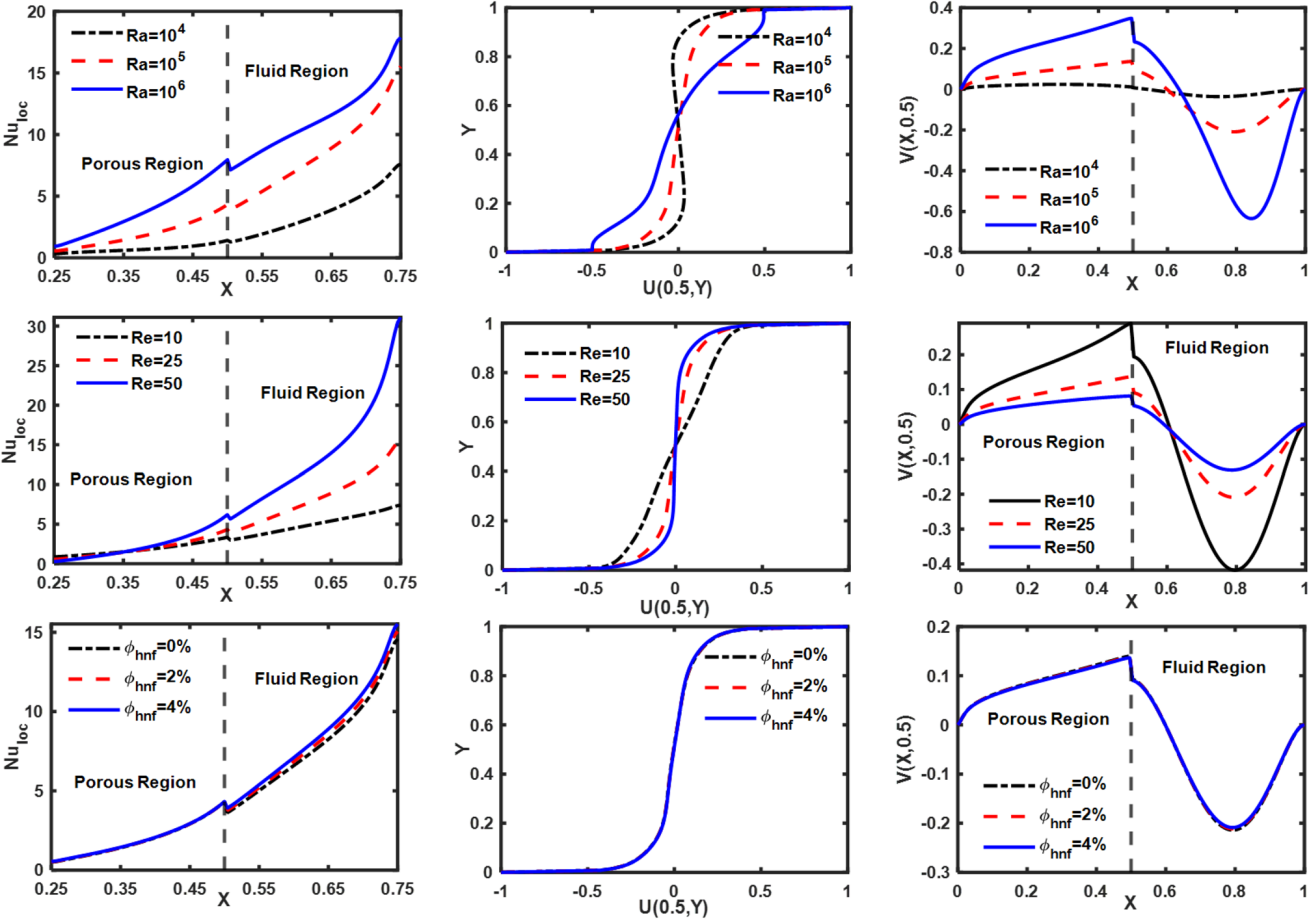
Influence of ‘Ra’, ‘Re’ and ‘$\phi_{{{\text{hnf}}}}$’ on velocity and Local Nusselt number profiles.

**Figure 13:**
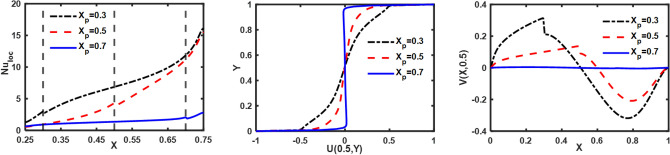
Influence of ‘X_p_’ on velocity and Local Nusselt number profiles.

The influence of Darcy number (Da), Hartmann number (Ha), and vortex viscosity (K_0_) on Nu_loc_; U(0.5,Y) and V(X, 0.5) is illustrated in Fig. 11. A reduction in “permeability of the porous medium” leads to a corresponding decrease in the Nu_loc_; U(0.5,Y) and V(X, 0.5). The existence of a magnetic field produces Lorentz force, which diminishes the movement of the fluid. Hence, with the rise in ‘Ha’ a decline in Nu_loc_; U(0.5,Y) and V(X, 0.5). Nu_loc_; U(0.5,Y) and V(X, 0.5) are reduced as the fluid's viscosity increases in response to an increase in the vortex viscosity parameter, as shown in Fig. 11. These results are consistent with the findings reported in the previous literature. Figure 12 demonstrates the influence of ‘Ra’, ‘Re’ and ‘$\phi_{{{\text{hnf}}}}$’ on Nu_loc_; U(0.5,Y) and V(X, 0.5). An increase in the Rayleigh number promotes the flow of fluid due to buoyancy forces, thereby enhancing the heat transfer from the heated wall. Consequently, Nu_loc_; U(0.5,Y), and V(X, 0.5) increase as ‘Ra’ increases. Increased wall motion directly correlates with increased suspension movement, resulting in a positive impact on thermal exchange. The rise in $\phi_{{{\text{hnf}}}}$ leads to an improvement in the thermal conductivity of the base fluid, thereby increasing the convection of heat. Furthermore, the influence of the thickness of the porous layer on Nu_loc_; U(0.5,Y) and V(X, 0.5) is depicted in Fig. 13. It is observed that an increase in the thickness of the porous layer leads to a decrease in heat convection from the heated wall as well as a reduction in flow velocity, i.e., U(0.5,Y) and V(X, 0.5). Table 4 presents the impact of different parameters on the “average Nusselt number (Nu_avg_), total entropy production (S_Total_), and average Bejan number (Be_avg_).”

The analysis of Table 4 provides the quantitative fluctuations in the “Nu_avg_ and S_Total_ in response to changes in dimensionless physical parameters. A higher ‘Ra’ value amplifies the buoyancy effect, thereby enhancing the heat transfer phenomenon within the fluid. The rise in ‘Ra’ from 10^4^ to 10^6^, the value of Nu_avg_ and S_Total_ upsurges by 244.96% and 285.97%, respectively. The rate of flow decreased as the Hartmann number raised due to the Lorentzian force, resulting in an upsurge in fluid temperature. Consequently, a reduction of 18.25% and 8.50% is observed in Nu_avg_ and S_Total_ respectively, as ‘Ha’ rises from 0 to 20. Augmenting the value of K_0_ leads to an elevation in dynamic viscosity, resulting in a reduction of heat convection. The Nu_avg_ drops by 25.25% as K_0_ increases from 2 to 8. Increasing K_0_ from 2 to 8 results in a significant 22.32% increase in S_Total_.

The addition of nanoparticles to a regular fluid improves its thermal properties, resulting in increased heat capacity and thermal conductivity, which causes heat convection to increase. As a result, boost in $\phi_{{{\text{hnf}}}}$ from 0 to 4%, a corresponding rise of 6.30% and 2.88% in Nu_avg_ and S_Total_. A decrease in 'Da' from 10^–3^ to 10^–5^ causes a reduction in permeability, leading to a 23.52% and 21.76% decrease in Nu_avg_ and S_Total_, respectively. Furthermore, an increase in the Reynolds number directly enhances the speed of the walls, thereby positively impacting the heat convection and entropy generation processes. Therefore, if ‘Re’ increases from 10 to 50, Nu_avg_ will increase by 134.94% and S_Total_ by 28.99%. The drag force caused by a porous media increases linearly with its thickness (X_p_) which leads to an increase in temperature within the enclosure. Therefore, as the thickness of the porous layer (X_p_) increases, heat convection and entropy production both decrease. Hence, Nu_avg_ and S_Total_ drop 410.02% and 266.75%, respectively, as X_p_ rises from 0.3 to 0.7.

## Conclusions

For a double lid-driven flow, this study investigates heat convection and entropy generation in a partially porous stratified hexagonal enclosure under the influence of a static magnetic field of strength ‘B_0_’. The hexagonal cavity was filled with a micropolar hybrid nanofluid that contained Ag and MgO nanoparticles, with water serving as the base fluid. The results were performed employing a range of values: 10^4^ $\le$ Ra $\le$ 10^6^, 0 $\le$ Ha $\le$ 20, $0.00 \le \phi_{{{\text{hnf}}}} \le 0.04$, 2.0 $\le$ K_0_ $\le$ 8.0, 10^–3^ $\le$ Da $\le$ 10^–5^, 10 $\le$ Re $\le$ 50, 0.3 $\le$ X_p_ $\le$ 0.7. Following the numerical results and discussions, the primary findings can be summarized as follows:

- An increase of ‘Ra’ from 10^4^ to 10^6^ results in a 244.96% and 285.97% improvement in Nu_avg_ and S_Total_, respectively, while an increase of ‘Ha’ from 0 to 20 causes an attenuation of 18.25% and 8.50% in Nu_avg_ and S_Total_.
- An upsurge in ‘K_0_’ from 2 to 8 triggers a decrease of 25.25% in ‘Nu_avg_’ and a 22.33% boost in S_Total_ because a rise in dynamic viscosity causes higher resistance to fluid motion.
- An increase in the ‘Re’ directly enhances the speed of the walls, thereby positively impacting the heat convection and entropy generation processes. Therefore, as ‘Re’ increases from 10 to 50, Nu_avg_ increases by 134.94% and S_Total_ by 28.99%.
- Nu_avg_ and S_Total_ decrease by 18.25% and 8.50%, respectively, as the ‘Ha’ increases from 0 to 20.
- As the porous layer thickness (X_p_) goes up from 0.3 to 0.7, Be_avg_ goes up by 16.695%, however a 410.02% and 266.75% reduction in Nu_avg_ and S_Total_ is noticed.
- The thermal conductivity of the base fluid is improved as $\phi_{{{\text{hnf}}}}$ rises from 0 to 4%, leading to a corresponding rise of 6.30% and 2.88% in Nu_avg_ and S_Total_.
- A decrease in ‘Da’ from 10^–3^ to 10^–5^ causes a reduction in permeability, leading to a 23.52% and 21.76% decrease in Nu_avg_ and S_Total_, respectively.
- The prevalence of frictional irreversibility becomes more pronounced as the values of ‘Ha’ and ‘K_0_’ go up, surpassing the prevalence of thermal irreversibility.

In conclusion, this study suggests that decreasing the vortex viscosity parameter (K_0_) can enhance heat transfer efficiency while simultaneously reducing entropy production. However, when the values of Ra, Re, and $\phi_{{{\text{hnf}}}}$ are increased, it leads to an enhancement in heat convection and entropy production. Conversely, an increase in Ha and X_p_ leads to a reduction in heat convection and entropy production.

## Data Availability

The datasets used and/or examined during the current study are available from the corresponding author on reasonable request.

## Appendix-1

1. The central finite difference method is employed on stream function equations, the resulting discretized form in porous layer and hybrid nanofluid layer are:
  $${\uppsi }_{{{\text{p}},{\text{ hnf}}}} \left( {{\text{i}},{\text{ j}}} \right) = \frac{1}{4}\left[ {{\text{h}}^{2} {\upomega }_{{{\text{p}},{\text{ hnf}}}} \left( {{\text{i}},{\text{ j}}} \right) + {\uppsi }_{{{\text{p}},{\text{hnf}}}} \left( {{\text{i}} + 1,{\text{ j}}} \right) + {\uppsi }_{{{\text{p}},{\text{hnf}}}} \left( {{\text{i}} - 1,{\text{ j}}} \right) + {\uppsi }_{{{\text{p}},{\text{hnf}}}} \left( {{\text{i}},{\text{ j}} + 1} \right) + {\uppsi }_{{{\text{p}},{\text{hnf}}}} \left( {{\text{i}},{\text{ j}} - 1} \right)} \right]$$
2. The central finite difference method is employed on vorticity equations, the resulting discretized form in porous layer and hybrid nanofluid layer are:
  $$\begin{aligned} {{\omega }}_{{{\text{p}},{\text{~hnf}}}} \left( {{\text{i}},{\text{~j}}} \right) & = \frac{1}{{{\text{A}}_{6} }}\left[ { - {\text{C}}_{{11}} + 4{\text{A}}_{1} {\text{C}}_{{12}} - 4{\text{A}}_{2} {\text{C}}_{{13}} + \frac{{2{\text{A}}_{4} }}{{\text{h}}}{\text{C}}_{{14}} + 4{\text{A}}_{5} {\text{C}}_{{15}} } \right] \\ {\text{A}}_{1} & {{ = \varepsilon ~}}\left( {\frac{1}{{{\text{Re}}}}} \right)\left( {\frac{{{{\rho }}_{{\text{f}}} }}{{{{\rho }}_{{{\text{hnf}}}} }}} \right)\left( {\frac{1}{{\left( {1 - \phi _{{{\text{hnf}}}} } \right)^{{2.5}} }} + {\text{K}}_{0} } \right){\text{;}}\;\;{\text{A}}_{2} = {{\varepsilon }}^{2} {\text{K}}_{0} {\text{~}}\left( {\frac{{{{\rho }}_{{\text{f}}} }}{{{{\rho }}_{{{\text{hnf}}}} }}} \right)\left( {\frac{1}{{{\text{Re}}}}} \right);~\;\;{\text{A}}_{3} = {{\varepsilon }}^{2} {{\delta }}\left( {\frac{1}{{{\text{Re~Da}}}}} \right)\left( {\frac{{{{\rho }}_{{\text{f}}} }}{{{{\rho }}_{{{\text{hnf}}}} }}} \right)\left( {\frac{1}{{\left( {1 - \phi _{{{\text{hnf}}}} } \right)^{{2.5}} }}} \right); \\ {\text{A}}_{4} & = {{\varepsilon }}^{2} \left( {\frac{{{\text{Ra}}}}{{{\text{Re}}^{2} {\text{Pr}}}}} \right)\frac{{\left( {{{\rho \beta }}} \right)_{{{\text{hnf}}}} }}{{{{\rho }}_{{{\text{hnf}}}} {{~\beta }}_{{\text{f}}} }};\;{\text{A}}_{5} = {{\varepsilon }}^{2} \frac{{{\text{Ha}}^{2} }}{{{\text{Re}}}}\left( {\frac{{{{\rho }}_{{\text{f}}} }}{{{{\rho }}_{{{\text{hnf}}}} }}} \right)\left( {\frac{{{{\sigma }}_{{{\text{hnf}}}} }}{{{{\sigma }}_{{\text{f}}} }}} \right)~;\;{\text{A}}_{6} = 16{\text{A}}_{1} {\text{ + 4A}}_{3} {\text{h}}^{2} \\ \end{aligned}$$

The coefficients C_1i_ where i = 1–4, and defined below:

$$\begin{aligned} {\text{C}}_{11} & = \left( {{\uppsi }_{{{\text{p}},{\text{ hnf}}}} \left( {{\text{i}},{\text{ j}} + 1} \right) - {\uppsi }_{{{\text{p}},{\text{ hnf}}}} \left( {{\text{i}},{\text{ j}} - 1} \right)} \right)\left( {{\upomega }_{{{\text{p}},{\text{ hnf}}}} \left( {{\text{i}} + 1,{\text{ j}}} \right) - {\upomega }_{{{\text{p}},{\text{ hnf}}}} \left( {{\text{i}} - 1,{\text{ j}}} \right)} \right) \\ & \;\; - \left( {{\uppsi }_{{{\text{p}},{\text{ hnf}}}} \left( {{\text{i}} + 1,{\text{ j}}} \right) - {\uppsi }_{{{\text{p}},{\text{ hnf}}}} \left( {{\text{i}} - 1,{\text{ j}}} \right)} \right)\left( {{\upomega }_{{{\text{p}},{\text{ hnf}}}} \left( {{\text{i}},{\text{ j}} + 1} \right) - {\upomega }_{{{\text{p}},{\text{ hnf}}}} \left( {{\text{i}},{\text{ j}} - 1} \right)} \right) \\ \end{aligned}$$

$${\text{C}}_{12} = \left( {{\upomega }_{{{\text{p}},{\text{ hnf}}}} \left( {{\text{i}} + 1,{\text{ j}}} \right) + {\upomega }_{{{\text{p}},{\text{ hnf}}}} \left( {{\text{i}} - 1,{\text{ j}}} \right) + {\upomega }_{{{\text{p}},{\text{ hnf}}}} \left( {{\text{i}},{\text{ j}} + 1} \right) + {\upomega }_{{{\text{p}},{\text{ hnf}}}} \left( {{\text{i}},{\text{ j}} - 1} \right)} \right)$$

$${\text{C}}_{13} = \left( {{\text{M}}_{{{\text{p}},{\text{ hnf}}}} \left( {{\text{i}} + 1,{\text{ j}}} \right) + {\text{M}}_{{{\text{p}},{\text{ hnf}}}} \left( {{\text{i}} - 1,{\text{ j}}} \right) + {\text{M}}_{{{\text{p}},{\text{ hnf}}}} \left( {{\text{i}},{\text{ j}} + 1} \right) + {\text{M}}_{{{\text{p}},{\text{ hnf}}}} \left( {{\text{i}},{\text{ j}} - 1} \right) - 4{\text{M}}_{{{\text{p}},{\text{ hnf}}}} \left( {{\text{i}},{\text{ j}}} \right){ }} \right){ }$$

$${\text{C}}_{14} = \left( {{\uptheta }_{{{\text{p}},{\text{hnf}}}} \left( {{\text{i}} + 1,{\text{ j}}} \right) - {\uptheta }_{{{\text{p}},{\text{ hnf}}}} \left( {{\text{i}} - 1,{\text{ j}}} \right)} \right)$$

$${\text{C}}_{15} = \left( {{\uppsi }_{{{\text{p}},{\text{ hnf}}}} \left( {{\text{i}} + 1,{\text{ j}}} \right) - 2{\uppsi }_{{{\text{p}},{\text{ hnf}}}} \left( {{\text{i}},{\text{j}}} \right) + {\uppsi }_{{{\text{p}},{\text{ hnf}}}} \left( {{\text{i}} - 1,{\text{ j}}} \right)} \right)$$

3. On employing central finite difference method, the angular momentum equation in discretized form for porous layer and hybrid nanofluid layer are:
  $${\text{M}}_{{{\text{p}},{\text{ hnf}}}} \left( {{\text{i}},{\text{ j}}} \right) = \frac{1}{{{\text{A}}_{9} }}\left[ { - {\text{D}}_{11} + 4{\text{A}}_{7} {\text{D}}_{12} + 4{\text{A}}_{8} {\text{h}}^{2} {\upomega }_{{{\text{p}},{\text{hnf}}}} \left( {{\text{i}},{\text{ j}}} \right)} \right]$$
  $${\text{A}}_{{7}} {{ = \varepsilon }}\left( {\frac{1}{{{\text{Re}}}}} \right)\left( {\frac{{{\uprho }_{{\text{f}}} }}{{{\uprho }_{{{\text{hnf}}}} }}} \right)\left( {\frac{1}{{\left( {1 - \phi_{{{\text{hnf}}}} } \right)^{2.5} }} + \frac{{{\text{K}}_{0} }}{2}} \right);{\text{A}}_{{8}} \, = \,\chi 2{\varepsilon K}_{0} \left( {\frac{{{\uprho }_{{\text{f}}} }}{{{\uprho }_{{{\text{hnf}}}} }}} \right)\left( {\frac{1}{{{\text{Re}}}}} \right);{\text{A}}_{{9}} \, = \,{\text{16A}}_{{7}} \, + \,{\text{8A}}_{{8}}$$
  $$\begin{aligned} {\text{D}}_{11} & = \left( {{\uppsi }_{{{\text{p}},{\text{hnf}}}} \left( {{\text{i}},{\text{ j}} + 1} \right) - {\uppsi }_{{{\text{p}},{\text{hnf}}}} \left( {{\text{i}},{\text{ j}} - 1} \right)} \right)\left( {{\text{M}}_{{{\text{p}},{\text{hnf}}}} \left( {{\text{i}} + 1,{\text{ j}}} \right) - {\text{M}}_{{{\text{p}},{\text{hnf}}}} \left( {{\text{i}} - 1,{\text{ j}}} \right)} \right) - \left( {{\uppsi }_{{{\text{p}},{\text{hnf}}}} \left( {{\text{i}} + 1,{\text{ j}}} \right) - {\uppsi }_{{{\text{p}},{\text{hnf}}}} \left( {{\text{i}} - 1,{\text{ j}}} \right)} \right) \\ & \;\;\;\left( {{\text{M}}_{{{\text{p}},{\text{hnf}}}} \left( {{\text{i}},{\text{ j}} + 1} \right) - {\text{M}}_{{{\text{p}},{\text{hnf}}}} \left( {{\text{i}},{\text{ j}} - 1} \right)} \right). \\ \end{aligned}$$
  $${\text{D}}_{12} = \left( {{\text{M}}_{{{\text{p}},{\text{hnf}}}} \left( {{\text{i}} + 1,{\text{ j}}} \right) + {\text{M}}_{{{\text{p}},{\text{hnf}}}} \left( {{\text{i}} - 1,{\text{ j}}} \right) + {\text{M}}_{{{\text{p}},{\text{hnf}}}} \left( {{\text{i}},{\text{ j}} + 1} \right) + {\text{M}}_{{{\text{p}},{\text{hnf}}}} \left( {{\text{i}},{\text{ j}} - 1} \right)} \right)$$
4. The discretized form of the energy equation in porous layer and hybrid nanofluid layer after implementing central finite difference method are: ${\uptheta }_{{{\text{p}},{\text{hnf}}}} \left( {{\text{i}},{\text{ j}}} \right) = \frac{1}{{{\text{A}}_{10} }}\left[ { - {\text{T}}_{11} + {\text{A}}_{11} {\text{T}}_{12} } \right]$; where, ${\text{A}}_{10} = \frac{{16 {\upalpha }^{*} }}{{{\upalpha }_{{\text{f}}} }}$; ${\text{A}}_{11} = \frac{{4 {\upalpha }^{*} }}{{{\upalpha }_{{\text{f}}} }}$.

The porous layer and clear fluid zone conform to: ${\upvarepsilon } = 1$, ${\updelta } = 0$, ${\upalpha }^{*} = {\upalpha }_{{{\text{hnf}}}}$ in fluid region and ${\upvarepsilon } = {\upvarepsilon }$, ${\updelta } = 1$, ${\upalpha }^{*} = {\upalpha }_{{{\text{eff}}}}$, in porous stratum.

$$\begin{aligned} {\text{T}}_{11} & = \left( {{\uppsi }_{{{\text{p}},{\text{hnf}}}} \left( {{\text{i}},{\text{ j}} + 1} \right) - {\uppsi }_{{{\text{p}},{\text{hnf}}}} \left( {{\text{i}},{\text{ j}} - 1} \right)} \right)\left( {{\uptheta }_{{{\text{p}},{\text{hnf}}}} \left( {{\text{i}} + 1,{\text{ j}}} \right) - {\uptheta }_{{{\text{p}},{\text{hnf}}}} \left( {{\text{i}} - 1,{\text{ j}}} \right)} \right) \\ & \;\;\; - \left( {{\uppsi }_{{{\text{p}},{\text{hnf}}}} \left( {{\text{i}} + 1,{\text{ j}}} \right) - {\uppsi }_{{{\text{p}},{\text{hnf}}}} \left( {{\text{i}} - 1,{\text{ j}}} \right)} \right)\left( {{\uptheta }_{{{\text{p}},{\text{hnf}}}} \left( {{\text{i}},{\text{ j}} + 1} \right) - {\uptheta }_{{{\text{p}},{\text{hnf}}}} \left( {{\text{i}},{\text{ j}} - 1} \right)} \right) \\ \end{aligned}$$

$${\text{T}}_{12 = } \left( {{\uptheta }_{{{\text{p}},{\text{hnf}}}} \left( {{\text{i}} + 1,{\text{ j}}} \right) + {\uptheta }_{{{\text{p}},{\text{hnf}}}} \left( {{\text{i}} - 1,{\text{ j}}} \right) + {\uptheta }_{{{\text{p}},{\text{hnf}}}} \left( {{\text{i}},{\text{ j}} + 1} \right) + {\uptheta }_{{{\text{p}},{\text{hnf}}}} \left( {{\text{i}},{\text{ j}} - 1} \right)} \right)$$

The impermeable interface conditions are numerically expressed by taking three points backward and forward gradients for porous and hybrid nanofluid layers, respectively. As a result, the interface potentials are computed using the following difference equations:

$${\uppsi }_{{{\text{int}}}} \left( {{\text{i}},{\text{ j}}} \right) = \frac{{4{\uppsi }_{{{\text{hnf}}}} \left( {{\text{i}},{\text{ j}} + 1} \right) - {\uppsi }_{{{\text{hnf}}}} \left( {{\text{i}},{\text{j}} + 2} \right) + 4{\uppsi }_{{\text{p}}} \left( {{\text{i}},{\text{ j}} - 1} \right) - {\uppsi }_{{\text{p}}} \left( {{\text{i}},{\text{j}} - 2} \right)}}{6}$$

$${\upomega }_{{{\text{int}}}} \left( {{\text{i}},{\text{ j}}} \right) = \frac{{4{\upomega }_{{{\text{hnf}}}} \left( {{\text{i}},{\text{ j}} + 1} \right) - {\upomega }_{{{\text{hnf}}}} \left( {{\text{i}},{\text{j}} + 2} \right) + 4{\upomega }_{{\text{p}}} \left( {{\text{i}},{\text{ j}} - 1} \right) - {\upomega }_{{\text{p}}} \left( {{\text{i}},{\text{j}} - 2} \right)}}{6}$$

$${\text{N}}_{{{\text{int}}}} \left( {{\text{i}},{\text{ j}}} \right) = \frac{{4{\text{M}}_{{{\text{hnf}}}} \left( {{\text{i}},{\text{ j}} + 1} \right) - {\text{M}}_{{{\text{hnf}}}} \left( {{\text{i}},{\text{j}} + 2} \right) + 4{\text{M}}_{{\text{p}}} \left( {{\text{i}},{\text{ j}} - 1} \right) - {\text{M}}_{{\text{p}}} \left( {{\text{i}},{\text{j}} - 2} \right)}}{6}$$

$${\uptheta }_{{{\text{int}}}} \left( {{\text{i}},{\text{ j}}} \right) = \frac{{4{\uptheta }_{{{\text{hnf}}}} \left( {{\text{i}},{\text{ j}} + 1} \right) - {\uptheta }_{{{\text{hnf}}}} \left( {{\text{i}},{\text{j}} + 2} \right) + {\text{kr}}\left( {4{\uptheta }_{{\text{p}}} \left( {{\text{i}},{\text{ j}} - 1} \right) - {\uptheta }_{{\text{p}}} \left( {{\text{i}},{\text{j}} - 2} \right)} \right)}}{{3\left( {1 + {\text{k}}_{{{\text{eff}}}} } \right)}}$$

Here subscripts ‘int’ denotes the interface and ${\text{kr}} = \frac{{{\text{k}}_{{{\text{eff}}}} }}{{{\text{k}}_{{{\text{hnf}}}} }}.$

## List of symbols

${\text{B}}_{0}$: Magnetic field intensity (T)
${\text{Be}}_{{{\text{avg}}}}$: Average Bejan number
${\text{C}}_{{\text{p}}}$: Specific isobaric heat per unit mass (${\text{Jkg}}^{ - 1} {\text{K}}^{ - 1}$)
${\text{K}}_{0}$: Dimensionless Vortex viscosity parameter
${\text{Nu}}_{{{\text{avg}}}}$: Average Nusselt number
${\text{Nu}}_{{\text{loc }}}$: Local Nusselt number
${\text{S}}_{{{\text{loc}}}}$: Dimensionless local entropy generation
${\text{X}}_{{\text{p}}}$: Thickness of porous stratum
Be_local_: Local Bejan Number
Da: Darcy Number
FDM: Finite Difference Method
$${\text{g}}$$: Gravitational acceleration ($${\text{m s}}^{ - 2}$$)
${\text{H}}$: Length and width of square cavity (${\text{m}}$)
Ha: Hartmann number
HNFs: Hybrid Nanofluids
${\text{J}}$: Micro-inertial density (${\text{m}}^{ - 2}$)
$${\text{k}}$$: Thermal conductivity ($${\text{Wm}}^{ - 1} {\text{K}}^{ - 1} )$$
${\text{K}}$: Permeability of porous medium
${\text{M}}$: Dimensionless micro-rotation vector normal to xy-plane
MFs: Micropolar Fluid
NFs: Nanofluids
$${\text{P}}$$: Dimensionless pressure
$${\text{p}}$$: Pressure ($${\text{Pa}}$$)
$${\text{Pr}}$$: Prandtl number
$${\text{Ra}}$$: Rayleigh number
Re: Reynolds number
STotal: Total Entropy generation
$${\text{T}}$$: Dimensional temperature ($${\text{K}}$$)
${\text{u}},{\text{ U}}$: Dimensional and dimensionless velocity along x-axis (${\text{m s}}^{ - 1}$)s
${\text{v}},{\text{ V}}$: Dimensional and Dimensionless velocity along y-axis (${\text{m s}}^{ - 1}$)
${\text{X}},{\text{ Y }}$: Dimensionless Cartesian co-ordinates
${\text{x}},{\text{y }}$: Dimensional Cartesian co-ordinates (m)
$${\upvarepsilon }$$: Porosity
κ: Vortex viscosity parameter

## Greek symbols

$$\mathrm{\alpha }$$: Thermal diffusivity ($${{\text{m}}}^{2}{{\text{s}}}^{-1}$$)
$\upbeta$: Volumetric thermal expansion coefficient (${{\text{K}}}^{-1}$)
$\upgamma$: Spin gradient viscosity
$$\upmu$$: Dynamic viscosity $$({{\text{Kgm}}}^{-1}{{\text{s}}}^{-1}$$)
$$\upnu$$: Kinematic viscosity ($${{\text{m}}}^{-2}{{\text{s}}}^{-1}$$ )
$$\uprho$$: Density ($${\text{Kg}}{\mathrm{ m}}^{-3}$$)
$$\upphi$$: Volume fraction
$\upomega$: Dimensionless vorticity function
${\uppsi }$: Dimensionless Stream function
$${\uptheta }$$: Dimensionless temperature
$${\upchi }$$: Material parameter

## Subscripts

p: Porous medium
$${\text{avg}}$$: Average
$${\text{f}}$$: Base fluid
$${\text{hnf}}$$: Hybrid nanoflui
eff: Effective
$${\text{Ag}}$$: Silver
$${\text{MgO}}$$: Magnesium Oxide
